# A repository of the salivary metabolome and its key drivers in 1436 European children

**DOI:** 10.1016/j.ebiom.2025.106019

**Published:** 2025-11-11

**Authors:** Ellen De Paepe, Emile Callemeyn, Kimberly De Windt, Kathleen Wijnant, Vera Plekhova, Beata Pomian, Pablo Vangeenderhuysen, Stefaan De Henauw, Nathalie Michels, Heli Viljakainen, Marja H. Leppänen, Timo A. Lakka, Karolien Van De Maele, Nele Baeck, Ruth De Bruyne, Sander Lefere, Anja Geerts, Matthijs Vynck, Lynn Vanhaecke

**Affiliations:** a Department of Translational Physiology, Infectiology and Public Health, Faculty of Veterinary Medicine, Ghent University, Laboratory of Integrative Metabolomics (LIMET), Belgium; b Department of Internal Medicine and Paediatrics, Faculty of Medicine and Health Sciences, Ghent University, Hepatology Research Unit, Belgium; c Department of Public Health and Primary Care, Faculty of Medicine and Health Sciences, Ghent University, Belgium; d Faculty of Medicine, University of Helsinki, Finland; e Folkhälsan Research Centre, Program for Public Health, Finland; f School of Medicine, Institute of Biomedicine, University of Eastern Finland, Finland; g Foundation for Research in Health Exercise and Nutrition, Kuopio Research Institute of Exercise Medicine, Finland; h Department of Clinical Physiology and Nuclear Medicine, Kuopio University Hospital, Kuopio, Finland; i Department of Paediatrics, Antwerp University Hospital, Belgium; j Department of Paediatrics, Paediatric Gastroenterology, General Hospital Jan Palfijn Ghent, Belgium; k Department of Paediatric Gastroenterology, Hepatology and Nutrition, Ghent University Hospital, Liver Research Centre Ghent, Belgium; l Department of Gastroenterology & Hepatology, Ghent University Hospital, Liver Research Centre Ghent, Belgium; m School of Biological Sciences, Queens' University Belfast, United Kingdom

**Keywords:** Metabolomics, Paediatric health, Saliva, Obesity

## Abstract

**Background:**

Saliva is an emerging but understudied biofluid in metabolomics, offering a similar window into biological information as blood, while enabling repetitive and non-invasive sampling.

**Methods:**

In this observational study, we comprehensively analysed the salivary metabolome of 965 children and adolescents (7–17y) from two exploratory cohorts in Europe, using ultra-high performance liquid chromatography coupled to high-resolution mass spectrometry. Preprocessed data were assessed via (partial) Spearman correlation, Mummichog-based pathway enrichment, and univariate (Welsh t-test, one-way ANOVA, Wilcoxon or Kruskal–Wallis rank-sum test) analyses. All *p*-values were corrected using the Benjamini-Hochberg procedure. Associations with age, anthropometrics, mental wellbeing, lifestyle, dietary intake and microbiome were explored. Key metabolite associations were validated in two independent cohorts (8–18y, n = 471), yielding a total study population of 1436 participants.

**Findings:**

Over 3500 untargeted features and 188 targeted metabolites were consistently detected in the pooled quality control saliva of the exploratory cohorts. Weight status (|ρ| < 0.255) and age (|ρ| < 0.341) reflected the salivary metabolome best, compared to mental wellbeing (|ρ| < 0.227) and lifestyle factors (|ρ| < 0.185). Multiple metabolites, especially (acetylated) amino acids and derivatives were elevated in participants with overweight or obesity and those experiencing high psychological stress, mirroring blood-based studies. In addition, these metabolites correlated with Bacteroidota, Pseudomonadota, Actinomycetota and Bacillota (|ρ| < 0.423) phyla, suggesting oral microbiome disturbances. Notably, the artificial sweetener acesulfame K, detected for the first time in children and adolescents’ saliva, was associated with excess weight (|ρ| < 0.243), psychological stress (|ρ| < 0.148), specific food and/or beverage consumption (|ρ| < 0.348) and *Bacillota* spp. (|ρ| < 0.196).

**Interpretation:**

Our findings reinforce and expand upon previous blood-based studies, while revealing novel salivary biomarkers and, as such, provide a high-quality repository of the paediatric salivary metabolome for future studies exploring its association with children and adolescent's health.

**Funding:**

FAME and OPERA were supported by the Research Foundation Flanders, Ghent University, and the European Research Council. PANIC was funded by Finnish national agencies and foundations. Fin-HIT was funded by the Folkhälsan Research Foundation and the Päivikki and Sakari Sohlberg Foundation.

**Research in context**

**Evidence before this study**

Previous research has shown that the metabolome, influenced by genetics and external factors such as diet, lifestyle, and microbiome, is crucial for understanding complex diseases like obesity and depression. Saliva, as a non-invasive biofluid, offers a practical alternative to blood for metabolomics studies, especially in children and adolescents. However, past studies have faced reproducibility issues due to small sample sizes and non-optimised analytical methods. No comprehensive studies have yet explored and externally validated the key drivers of the salivary metabolome in relation to health in children and adolescents.

**Added value of this study**

This study provides a large-scale, comprehensive analysis of the salivary metabolome in children and adolescents, revealing both known – (acetylated) amino acids and derivatives – and novel biomarkers – acesulfame K – associated with weight status and mental wellbeing. These findings were validated in independent cohorts, enhancing the robustness and applicability of saliva for paediatric health monitoring.

**Implications of all the available evidence**

The results of this study demonstrate that saliva is a valuable non-invasive biofluid for monitoring paediatric health. This research provides new insights into the metabolic profiles associated with age, anthropometrics, mental wellbeing, and lifestyle factors, and how these profiles are related to dietary habits and microbial strains. By establishing a comprehensive salivary repository, our study enables future research to explore children and adolescents' health in greater depth. This repository paves the way for early diagnosis and personalised interventions, leveraging the salivary metabolome's association with health outcomes to improve paediatric medicine.

## Introduction

The metabolome represents a functional quantifiable readout of the human phenotype, reflecting metabolic activities within different cells, tissues, organs, and body fluids.^1^ As a result, it is unique and dynamic, driven by an individual's genome and exposome. The latter constitutes all external drivers an individual is exposed to, comprising mainly dietary, drug and supplement intake, psychosocial environment, lifestyle including physical activity (PA) and sleep, and the gastrointestinal microbiome.^2^ Unravelling the extent to which these various drivers affect the human metabolome is crucial for understanding the molecular origins of and the (patho)physiological pathways in which relevant metabolites can be mapped. This should ultimately enable correlating a particular metabotype to phenotypes of health and disease.^3^, ^4^, ^5^ Since metabolic alterations can be observed long before clinical manifestations, metabolomics facilitates both the elucidation of early-onset metabolic alterations and the prediction of disease susceptibility and prognosis.^6^

In recent years, metabolomics has contributed to the understanding of multiple complex diseases including obesity,^5^^,^^7^ depression,^8^^,^^9^ type 2 diabetes,^10^ cardiovascular disease^11^ and multiple cancers.^12^ These and other non-communicable diseases (NCDs) account for 43 million deaths annually, representing 75% of all deaths worldwide.^13^ It is worth pointing out that several NCDs often arise in early childhood, when healthy growth and development are crucial.^6^^,^^7^^,^^14^ Specifically, more than 400 million children and adolescents are currently suffering from overweight or obesity.^15^ Increasing levels of obesity are associated with reduced health-related quality of life,^16^ while posing a risk factor for the development of other NCDs and ultimately to premature death in adulthood.^17^ Therefore, it is particularly important to study the metabolome of children and adolescents and its main determinants, taking into account exposures in early life that may trigger the biological mechanism of diseases.^18^ This will not only allow for early diagnosis and prognosis but also pave the way towards the design of personalised intervention strategies promoting a healthy metabolome.^1^

The results of metabolomics studies have proven difficult to reproduce due to the personalised nature of the metabolome^1^^,^^7^ and small cohort sizes,^19^, ^20^, ^21^, ^22^, ^23^ highlighting the need for large cohort studies. Although urine has demonstrated its potential in studying different NCDs,^7^^,^^24^ there is some inconvenience with on-site and repeated sampling. For both clinical and metabolomics applications, blood plasma and serum are popular because they reflect systemic changes well.^25^ In vulnerable populations such as children, however, blood sampling is less feasible because it is considered painful, stressful and requires trained personnel. In this context, saliva is a valuable blood surrogate.^26^ Its painless, inexpensive and non-invasive nature of sample collection increases readiness and ensures compliance with large-scale and long-term studies.^26^^,^^27^ Since the oral cavity is host to both microbial and viral cells, the salivary metabolome comprises exogenous and endogenous metabolic products, the latter by reflecting circulating metabolite levels via passive diffusion from blood.^27^^,^^28^ Saliva thus presents superior properties to blood, as it provides a mirror of the interactions between the microbiome, diet and host metabolome.^26^^,^^27^^,^^29^ To the best of our knowledge, for the salivary metabolome, no studies report on its key drivers in relation to health yet.

In this cross-sectional study, we are the first to comprehensively characterize the salivary metabolome of children and adolescents, analysing samples from 1436 participants across four independent European paediatric cohorts (two from Belgium, two from Finland). Our main objectives were to unravel the extent to which the salivary metabolome reflects age and anthropometric parameters, particularly weight status, as well as indicators of mental wellbeing and lifestyle, and how food consumption and microbial composition contribute to that.

## Methods

### Paediatric cohorts

**Fig. 1:**
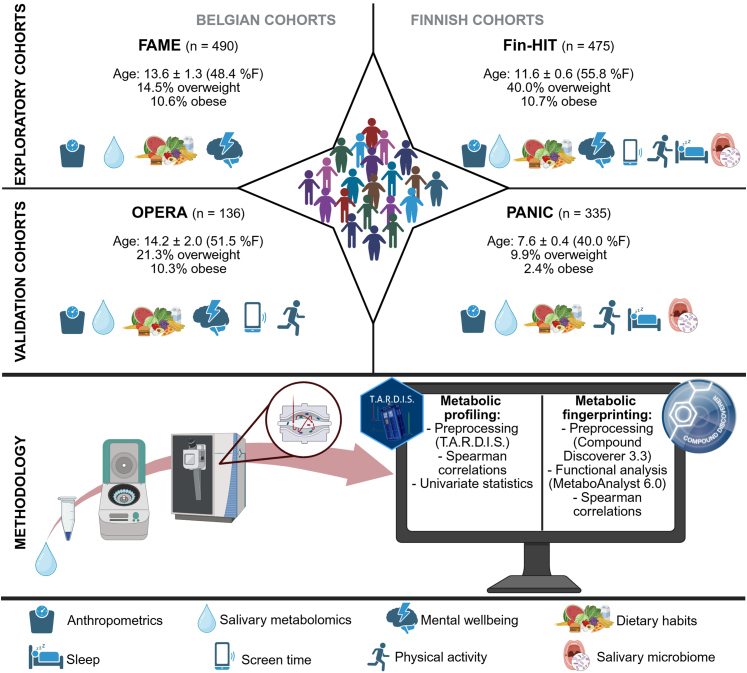
Schematic study design. Four different cohorts totalling 1436 children or adolescents from whom one saliva sample was obtained for metabolomics analyses according to Wijnant et al.^27^ Where marked, age (mean ± SD), sex (F = female), weight category, mental wellbeing, lifestyle (including sleep duration, screen time and physical activity), dietary habits and salivary microbiome data were collected. Classification of overweight and obesity was based on Cole et al.^30^

An overview of the different cohorts and collected data can be consulted in Fig. 1. The FAME (Flemish Adolescent MEtabolome)^31^ and Fin-HIT (Finnish Health In Teens)^32^ cohorts served as exploratory cohorts, while data from the PANIC (Physical Activity and Nutrition in Children)^33^^,^^34^ and OPERA (Obesity Prevention through Emotion Regulation in Adolescents)^35^ cohorts were utilised for independent validation.

Briefly, the FAME^31^ study (ClinicalTrials.gov NCT06391671) was conducted in both school and obesity clinic settings, with cross-sectional data collection taking place between February 2022 and May 2023. Anthropometric measurements, including height, weight, waist circumference, and body fat percentage (BF%), were taken using standardised methods by a trained professional, and BMI was calculated and adjusted for age and sex according to Belgian reference standards (BMI*z*).^36^ Personal data were collected alongside information on mental wellbeing and food intake through online self-report questionnaires. Stress perception was assessed using the Perceived Stress Scale (PSS), a 10-item instrument to evaluate the degree to which an individual has perceived life as unpredictable, uncontrollable and overloading over the previous month.^37^^,^^38^ Depressive symptoms were monitored with the Children's Depression Inventory (CDI-2), a 28-item questionnaire that is a validated instrument for measuring cognitive, affective, and behavioural indicators of depression in children and adolescents aged 8–21 years.^39^ Self-esteem was measured using the Rosenberg Self-Esteem Scale (RSES), a validated 10-item questionnaire to assess participants' self-esteem and perceived self-worth.^40^ Eating behaviour (external, restrained and emotional eating) was recorded using the 33-item Dutch Eating Behaviour Questionnaire (DEBQ),^41^^,^^42^ while dietary patterns were assessed with a 59-item food frequency questionnaire (FFQ) adapted from the Children's Eating Habits Questionnaire, designed to assess food consumption frequencies associated with overweight, obesity, and general health in the IDEFICS project.^43^^,^^44^ At least one guardian and the child jointly reported the frequency of the participant's consumption of selected food items during a typical week in the preceding 4 weeks, using the following response options: ‘(almost) never’, ‘a few times a week’, ‘(almost) daily’, or ‘multiple times per day’. These frequency categories were subsequently assigned numerical values of 0, 1, 2, and 3, respectively, without portion size quantification (Table S1).

The Fin-HIT^32^ study was conducted between 2011 and 2014, primarily in a school setting. Trained fieldworkers measured the participants’ weight, height and waist circumference using standardised methods. BMI scores were calculated and adjusted using the age- and sex-specific reference values of the International Obesity Task Force criteria (IOTF) to obtain the BMI*z*.^30^ Self-esteem was measured using the RSES,^40^ and body image (BI) was assessed using the Collins Childhood Body Rating Scales, a pictorial scale consisting of seven sex-specific images, representing a range of body sizes from very thin (1) to obese (7).^45^^,^^46^ Participants were asked to select the image that most resembled their current body shape and the one that reflected their desired body shape. The discrepancy between the current and desired body shape was then calculated as an indicator of body dissatisfaction. PA was assessed by self-reporting of weekly hours of leisure-time exercise, while sleep habits were reported separately for school and non-school days, including waking and bedtime hours to calculate sleep durations during weekdays and weekends separately. Screen time was evaluated using questions adapted from the WHO Health Behaviour in School-aged Children (HBSC) study.^47^ Dietary intake was evaluated using an FFQ, adapted from the HBSC study, that captured the consumption frequencies of 16 food and beverage items.^47^ Responses were recorded on a seven-point scale ranging from 0 (not consumed) to 6 (consumed several times per day), indicating the frequency of consumption for each item during the past month (Table S2).

The PANIC study^33^^,^^34^ (ClinicalTrials.gov NCT01803776) is being conducted at the Institute of Biomedicine, University of Eastern Finland, Kuopio Campus, Finland. Baseline data collection occurred between 2007 and 2009. Trained research staff collected anthropometric data, including BF%, height, weight and waist circumference, with BMI scores calculated and adjusted for age and sex according to Finnish reference standards (BMI*z*).^48^ Sleep duration was estimated using the Basic Nordic Sleep Questionnaire^49^ adapted for guardians to complete. PA was assessed using the PANIC Physical Activity and Sedentary Behaviour Questionnaire, which measured total PA separately for five weekdays and two weekend days.^50^ Total screen time was calculated by summing the use of different screens, such as TV, computer, game consoles, tablets, and mobile phones. Food consumption was measured using food records in grams per day, completed by parents over four consecutive days, consisting of two week and two weekend days, and was verified and analysed by clinical nutritionists using the Micro Nutrica® software (version 2.5, The Social Insurance Institution of Finland, Turku, Finland), incorporating food composition data from national analyses and international sources (Table S3).^51^

For the OPERA cohort^35^ cross-sectional data were collected between March and August 2018. Anthropometric measures, including height, weight, waist circumference, and BF%, were collected by a study nurse, and BMI scores were adjusted for age and sex (BMI*z*).^36^ Mental wellbeing was assessed using the PSS,^38^ CDI-2,^39^ the negative affect items of the Positive And Negative Affect Schedule for Children (PANAS-C)^52^ and hair cortisol. The PANAS-C consists of 30 self-report questions, to measure the participant's emotions during the past few weeks. Participants were asked to rate the extent to which they experienced each feeling over the past few weeks, using a 5-point Likert-type scale ranging from ‘very slightly or not at all’ to ‘extremely’. Eating behaviour (emotional and external eating) and food consumption were evaluated, using respectively the DEBQ^41^^,^^42^ and an FFQ^43^^,^^44^ as in the FAME (Table S1). Lifestyle data were collected through questionnaires assessing screen time and PA.

The included cohorts represent both population-based (PANIC, OPERA, Fin-HIT) and more dedicated (FAME) sampling strategies, enabling the investigation of salivary metabolite–phenotype associations across a broad spectrum of weight statuses. In Fin-HIT, a subset of participants with higher BMI*z* was selected, while FAME specifically oversampled participants with overweight and obesity. The percentage of missing metadata was generally low (< 10% in FAME and OPERA, < 1% in Fin-HIT, ≤ 5% PANIC, except FFQ which had < 20% missingness) (Table 1). Missing metadata were excluded from further analyses where applicable.

**Table 1:** Participants’ characteristics from the FAME, Fin-HIT, OPERA and PANIC cohorts.

	FAME			Fin-HIT			OPERA			PANIC		
***Participant numbers***		***N = 490***		***N = 466***			***N = 136***			***N = 335***		
*Boy/Girl*	253		237	210		256	66		70	201		134
*HW/OW/OB*	237	71	52	234	190	51	93	29	14	294	33	8
***Age (years)***			***N = 490***	***N = 466***			***N = 136***			***N = 335***		
*Boy/Girl*	13.50 ± 1.27		13.58 ± 1.29	11.59 ± 0.57		11.52 ± 0.53	14.47 ± 1.68		14.08 ± 2.16	7.66 ± 0.39		7.62 ± 0.37
*HW/OW/OB*	**13.44 ± 1.13**	**13.51 ± 1.44**	**14.25 ± 1.73∗**	11.58 ± 0.49	11.51 ± 0.60	11.58 ± 0.57	14.38 ± 1.72	14.21 ± 1.92	12.43 ± 3.06	**7.6 ± 0.38**	**7.80 ± 0.38**	**7.49 ± 0.37∗**
***Sex (%F)***			***N = 490***	***N = 466***			***N = 136***			***N = 335***		
*HW/OW/OB*	49.04%	42.25%	51.92%	58.12%	54.21%	50.98%	49.46%	41.38%	71.43%	39.79%	39.39%	50.00%
***Weight (kg)***			***N = 490***	***N = 466***			***N = 135***			***N = 335***		
*Boy/Girl*	54.35 ± 18.03		54.81 ± 16.35	47.07 ± 11.14		46.89 ± 10.52	61.05 ± 14.40		57.32 ± 12.89	27.10 ± 4.65		26.63 ± 5.14
*HW/OW/OB*	**47.30 ± 8.48**	**65.07 ± 11.39**	**91.58 ± 14.90∗**	**39.28 ± 5.63**	**51.47 ± 7.70**	**65.47 ± 10.21∗**	**53.80 ± 9.83**	**67.20 ± 10.50**	**78.15 ± 23.71∗**	**25.42 ± 3.31**	**34.56 ± 3.24**	**40.85 ± 4.83∗**
ρ (age)	**0.48∗**			**0.21∗**			<0.001			**0.241∗**		
***Height (m)***			***N = 490***	***N = 466***			***N = 135***			***N = 335***		
*Boy/Girl*	1.62 ± 0.11		1.61 ± 0.078	1.52 ± 0.087		1.52 ± 0.079	**1.70 ± 0.012**		**1.62 ± 0.096∗**	**1.30 ± 0.056**		**1.28 ± 0.057∗**
*HW/OW/OB*	**1.61 ± 0.095**	**1.61 ± 0.11**	**1.68 ± 0.093∗**	**1.50 ± 0.076**	**1.54 ± 0.077**	**1.57 ± 0.096∗**	**1.67 ± 0.011**	**1.65 ± 0.010**	**1.55 ± 0.016∗**	**1.28 ± 0.055**	**1.32 ± 0.051**	**1.33 ± 0.051∗**
ρ (age)	**0.60∗**			**0.33∗**			<0.001			**0.304∗**		
***BMIz***			***N = 490***	***N = 466***			***N = 136***			***N = 335***		
*Boy/Girl*	0.10 ± 1.29		0.24 ± 1.18	0.96 ± 0.90		0.82 ± 0.95	0.33 ± 1.21		0.48 ± 1.06	−0.22 ± 1.06		−0.16 ± 1.06
*HW/OW/OB*	**−0.40 ± 0.81**	**1.53 ± 0.31**	**2.38 ± 0.27∗**	**0.080 ± 0.55**	**1.48 ± 0.25**	**2.33 ± 0.29∗**	**−0.14 ± 0.59**	**1.44 ± 0.26**	**2.59 ± 0.44∗**	**−0.49 ± 0.86**	**1.45 ± 0.32**	**2.2 ± 0.20∗**
ρ (age)	0.066			−0.043			−0.144			0.024		
***Body fat percentage***			***N = 483***				***N = 133***			***N = 335***		
*Boy/Girl*	**20.12 ± 8.05**		**26.62 ± 7.36∗**	N.D.			**19.01 ± 7.61**		**27.12 ± 6.26∗**	**14.53 ± 6.43**		**18.29 ± 6.91∗**
*HW/OW/OB*	**19.78 ± 5.06**	**30.08 ± 6.46**	**38.61 ± 6.12∗**	N.D.			**19.98 ± 5.17**	**27.99 ± 6.54**	**40.51 ± 7.17∗**	**14.06 ± 5.08**	**27.56 ± 4.83**	**33.71 ± 4.29∗**
ρ (age)	<0.001			N.D.			<0.001			0.052		
***Waist circumference (cm)***			***N = 488***	***N = 464***			***N = 136***			***N = 335***		
*Boy/Girl*	**74.49 ± 13.60**		**71.78 ± 12.55∗**	**71.34 ± 8.97**		**68.49 ± 8.50∗**	74.87 ± 10.01		73.21 ± 10.01	**57.15 ± 5.17**		**56.24 ± 5.77∗**
*HW/OW/OB*	**66.93 ± 5.23**	**84.53 ± 7.72**	**101.58 ± 9.87∗**	**63.39 ± 4.04**	**73.50 ± 5.31**	**85.19 ± 8.23∗**	**69.51 ± 6.18**	**79.12 ± 5.70**	**94.68 ± 12.42∗**	**55.18 ± 3.51**	**65.54 ± 4.61**	**74.05 ± 3.72∗**
ρ (age)	<0.001			<0.001			**0.225∗**			0.135		
***Waist-to-height (cm/cm)***			***N = 488***	***N = 464***			***N = 135***			***N = 335***		
*Boy/Girl*	**0.46 ± 0.075**		**0.44 ± 0.072∗**	**0.47 ± 0.049**		**0.45 ± 0.050∗**	0.43 ± 0.052		0.45 ± 0.065	0.44 ± 0.035		0.44 ± 0.039
*HW/OW/OB*	**0.41 ± 0.028**	**0.52 ± 0.048**	**0.60 ± 0.050∗**	**0.42 ± 0.026**	**0.48 ± 0.035**	**0.54 ± 0.045∗**	**0.42 ± 0.027**	**0.49 ± 0.041**	**0.59 ± 0.060∗**	**0.43 ± 0.025**	**0.49 ± 0.037**	**0.55 ± 0.018∗**
ρ (age)	<0.001			<0.001			<0.001			−0.043		
***Obesity prevalence (%)***												
*Boy/Girl*	9.88%		11.37%	11.90%		9.81%	6.06%		17.14%	2.00%		2.98%
***Overweight prevalence (%)***												
*Boy/Girl*	16.20%		12.66%	41.43%		38.87%	25.75%		14.28%	9.95%		9.70%
***Psychological stress (z-score)***			***N = 443***	***N = 466***			***N = 131***					
*Boy/Girl*	**−0.57 ± 2.20**		**0.58 ± 2.72∗**	**0.75 ± 1.24**		**0.86 ± 1.05∗**	**−0.49 ± 2.72**		**0.51 ± 2.45∗**	N.D.		
*HW/OW/OB*	**−0.15 ± 2.56**	**0.36 ± 2.24**	**1.78 ± 2.49∗**	**0.63 ± 1.01**	**0.94 ± 1.23**	**1.17 ± 1.21∗**	−0.47 ± 2.47	0.80 ± 2.81	0.37 ± 1.75	N.D.		
ρ (age)	**0.16∗**			0.025			<0.001			N.D.		
***Perceived Stress Scale (PSS) (z-score)***			***N = 443***				***N = 131***					
*Boy/Girl*	**−0.20 ± 0.92**		**0.23 ± 0.98∗**	N.D.			**−0.21 ± 0.98**		**0.18 ± 1.01∗**	N.D.		
*HW/OW/OB*	**−0.089 ± 0.96**	**0.19 ± 0.92**	**0.59 ± 0.98∗**	N.D.			−0.15 ± 1.00	0.30 ± 1.05	−0.068 ± 0.78	N.D.		
ρ (age)	0.091			N.D.			<0.001			N.D.		
***Children Depression's Inventory-2 (CDI-2)*** ***(z-score)***			***N = 443***				***N = 131***					
*Boy/Girl*	**−0.20 ± 0.81**		**0.18 ± 1.08∗**	N.D.			−0.079 ± 1.07		0.10 ± 0.94	N.D.		
*HW/OW/OB*	**−0.12 ± 0.93**	**0.13 ± 0.86**	**0.67 ± 1.14∗**	N.D.			**−0.20 ± 0.96**	**0.34 ± 1.04**	**0.26 ± 0.88∗**	N.D.		
ρ (age)	**0.22∗**			N.D.			<0.001			N.D.		
***Rosenberg Self-Esteem Scale (RSES) (z-score)***			***N = 443***	***N = 466***								
*Boy/Girl*	**0.17 ± 0.86**		**−0.17 ± 1.06∗**	**0.23 ± 0.90**		**−0.19 ± 1.03∗**	N.D.			N.D.		
*HW/OW/OB*	**0.078 ± 0.99**	**−0.043** ± **0.90**	**−0.52 ± 0.83∗**	**0.11 ± 0.95**	**−0.075 ± 1.03**	**−0.25 ± 1.03∗**	N.D.			N.D.		
ρ (age)	**−0.16∗**			0.0080			N.D.			N.D.		
***Restrained eating (z-score)***			***N = 443***									
*Boy/Girl*	**−0.12 ± 0.93**		**0.13 ± 1.05∗**	N.D.			N.D.			N.D.		
*HW/OW/OB*	**−0.20 ± 0.92**	**0.74 ± 1.01**	**0.53 ± 0.90∗**	N.D.			N.D.			N.D.		
ρ (age)	<0.001			N.D.			N.D.			N.D.		
***Emotional eating (z-score)***			***N = 443***				***N = 133***					
*Boy/Girl*	**−0.17 ± 0.96**		**0.18 ± 1.01∗**	N.D.			−0.075 ± 1.12		0.090 ± 0.86	N.D.		
*HW/OW/OB*	**−0.067 ± 0.96**	**0.30 ± 1.08**	**0.098 ± 1.06∗**	N.D.			−0.066 ± 1.04	−0.088 ± 0.91	−0.47 ± 0.71	N.D.		
ρ (age)	<0.001			N.D.			<0.001			N.D.		
***External eating (z-score)***			***N = 443***				***N = 133***					
*Boy/Girl*	−0.050 ± 1.07		0.054 ± 0.91	N.D.			0.0072 ± 1.03		0.030 ± 0.95	N.D.		
*HW/OW/OB*	**0.054 ± 0.98**	**0.059 ± 1.05**	**−0.5 ± 0.92∗**	N.D.			**0.033 ± 0.95**	**−0.23 ± 1.10**	**−0.68 ± 1.26∗**	N.D.		
ρ (age)	<0.001			N.D.			<0.001			N.D.		
***Collins Childhood Body Rating Scale (z-score)***			***N = 466***									
*Boy/Girl*	N.D.			−0.080 ± 1.27		0.065 ± 0.71	N.D.			N.D.		
*HW/OW/OB*	N.D.			**0.45 ± 0.67**	**−0.34 ± 1.10**	**−0.79 ± 0.85∗**	N.D.			N.D.		
ρ (age)	N.D.			−0.043			N.D.			N.D.		
***Positive And Negative Affect Schedule for Children (PANAS-C) (z-score)***							***N = 131***					
*Boy/Girl*	N.D.			N.D.			**−0.2 ± 1.05**		**0.22 ± 0.94∗**	N.D.		
*HW/OW/OB*	N.D.			N.D.			−0.12 ± 0.98	0.17 ± 1.06	0.18 ± 0.72	N.D.		
ρ (age)	N.D.			N.D.			**0.177∗**			N.D.		
***Hair cortisol (z-score)***							***N = 127***					
*Boy/Girl*	N.D.			N.D.			**−0.28 ± 0.90**		**0.21 ± 0.94∗**	N.D.		
*HW/OW/OB*	N.D.			N.D.			−0.13 ± 0.95	0.11 ± 1.01	−0.22 ± 1.11	N.D.		
ρ (age)	N.D.			N.D.			<0.001			N.D.		
***Physical activity (hour/week)***				***N = 466***			***N = 133***			***N = 333***		
*Boy/Girl*	N.D.			6.78 ± 2.71		6.31 ± 2.74	4.80 ± 6.35		5.07 ± 5.30	**13.99 ± 5.17**		**12.35 ± 4.51∗**
*HW/OW/OB*	N.D.			6.58 ± 2.71	6.59 ± 2.67	5.94 ± 2.97	5.06 ± 6.20	3.99 ± 5.14	6.28 ± 8.60	12.76 ± 4.91	12.38 ± 4.40	12.55 ± 4.65
ρ (age)	N.D.			0.066			<0.001			<0.001		
***Screen time (hour/day)***				***N = 462***						***N = 333***		
*Boy/Girl*	N.D.			**4.02 ± 2.30**		**3.28 ± 2.03∗**	N.D.			**1.46 ± 0.77**		**1.87 ± 0.92∗**
*HW/OW/OB*	N.D.			**3.30 ± 2.09**	**3.74 ± 2.07**	**4.56** ± **2.63∗**	N.D.			1.69 ± 0.89	1.77 ± 0.83	1.94 ± 1.10
ρ (age)	N.D.			<0.001			N.D.			<0.001		
***Sleep quality week (z-score)***				***N*** = ***461***								
*Boy/Girl*	N.D.			0.030 ± 1.05		−0.023 ± 0.96	N.D.			N.D.		
*HW/OW/OB*	N.D.			0.067 ± 0.89	−0.048 ± 1.09	−0.14 ± 1.09	N.D.			N.D.		
ρ (age)	N.D.			<0.001			N.D.			N.D.		
***Sleep quality weekend (z-score)***				***N*** = ***461***								
*Boy/Girl*	N.D.			0.014 ± 1.02		−0.011 ± 0.98	N.D.			N.D.		
*HW/OW/OB*	N.D.			0.041 ± 0.95	−0.062 ± 1.06	0.043 ± 0.98	N.D.			N.D.		
ρ (age)	N.D.			<0.001			N.D.			N.D.		
***Sleep (hour/night)***										***N*** = ***318***		
*Boy/Girl*	N.D.			N.D.			N.D.			9.88 ± 0.58		9.84 ± 0.58
*HW/OW/OB*	N.D.			N.D.			N.D.			9.87 ± 0.62	9.80 ± 0.59	9.86 ± 0.58
ρ (age)	N.D.			N.D.			N.D.			<0.001		

HW = healthy weight; OW = overweight; OB = obese; N.D. = not determined.

Classification of overweight and obesity was based on Cole et al.^30^ Stress levels were determined using a psychological stress *z-*score. For the FAME cohort, the *z-*scores of CDI-2 and PSS were summed, and RSES subtracted (as higher RSES scores indicate better mental well-being). In the Fin-HIT cohort, the absolute value of the *z-*score-transformed Collins Childhood Body Rating Scale score was used, with RSES subtracted. For the PANIC cohort, only sleep was used. For the OPERA cohort, the *z-*scores of PSS, CDI-2, and negative PANAS-C were combined. Pairwise univariate comparisons were performed using the two-sided Welch's t-test for normally distributed data (Shapiro–Wilk *p* > 0.05 for all groups) or the two-sided Wilcoxon rank-sum test for non-normally distributed data (Shapiro–Wilk *p* ≤ 0.05 for at least one group). For multiple comparisons, either the one-way ANOVA (Shapiro–Wilk *p* > 0.05) or the two-sided Kruskal–Wallis rank-sum test (Shapiro–Wilk *p* ≤ 0.05) was used, followed by post hoc tests (Tukey or Dunn following ANOVA or Kruskal–Wallis). Age was evaluated using Spearman correlations.

Values are presented as mean ± standard deviation (SD) or percentage (%), with the number of participants with available data (N) indicated. Association with sex and weight class, and Spearman correlations (ρ) with age are shown. Significant results (∗*p* ≤ 0.05) are highlighted in bold.

### Salivary sample collection

All study participants were pseudonymised prior to sample and data collection. In the FAME cohort, saliva samples were collected in the morning at home by the study participant, using the passive drool collection method (Salimetrics, Carlsbad, USA), before any food or beverage consumption or teeth brushing. Samples were immediately stored at −12 to −20 °C for up to one week and then transferred under cooled conditions to the lab. For the Fin-HIT cohort, unstimulated saliva samples were collected using the Oragene® DNA Self-Collection Kit (Ontario, Canada) between 8 and 11 a.m., and stored at room temperature until further analysis. In the PANIC cohort, saliva was collected by a research nurse from 7 to 10 a.m. after an overnight fast. Participants chewed paraffin to stimulate saliva excretion, with saliva excreted over the first 30 s discarded, and the rest collected over the next 3 min. For the OPERA cohort, saliva was collected under the supervision of trained staff at the Ghent University Hospital using the passive drool collection method (Salimetrics, Carlsbad, USA) between 4.30 and 5.30 p.m. Participants were required to refrain from eating or drinking (except water) for at least 3 h before collection and could only brush their teeth in the morning. Smoking or alcohol use was not permitted during the whole day. Samples from the FAME, PANIC and OPERA cohorts were stored at −80 °C until analysis.

### Salivary metabolome analysis

Saliva samples were randomised, extracted and analysed per cohort using our validated salivary metabolomics method using ultra-high performance liquid chromatography quadrupole-Orbitrap high-resolution mass spectrometry (UHPLC-Q-Orbitrap-HRMS, Thermo Fisher Scientific, San José, CA, USA), as previously described by Wijnant et al., 2020.^27^ In short, 450 μL of saliva was pipetted into a 1.5 mL Eppendorf tube, followed by the addition of 10 μL of an internal standard mixture (Table S4). The solution was vortexed briefly and centrifuged for 5 min at 17,000 × *g* at room temperature. The supernatant was then collected with a 1 mL syringe and passed over a polyamide membrane filter (25 mm diameter, 0.45 mm pore size, Machery-Nagel, Germany). Finally, 150 μL of the undiluted extract was transferred to a UHPLC-vial with a glass insert.

UHPLC-HRMS analysis of the saliva extracts from the FAME and PANIC cohorts was performed in 2023 and 2022, respectively, using a Vanquish Horizon UHPLC system, coupled to an Orbitrap Exploris 120 MS (Thermo Fisher Scientific, San José, CA, USA), equipped with a heated electrospray ionization source (HESI-II) operating in polarity switching mode (position: interL/M/1.5). For the Fin-HIT and OPERA cohorts, analysed in 2021 and 2018, respectively, chromatographic separation was achieved using a Dionex Ultimate 3000 XRS UHPLC system, which was coupled to a Q-Exactive mass spectrometer (Thermo Fisher Scientific, San José, CA, USA), with a HESI-II source in polarity switching mode and positioned in 0/B/1. Both instruments were equipped with an Acquity HSS T3 C18 column (1.8 mm, 150 × 2.1 mm) (Waters, Manchester, UK) maintained at 45 °C. The binary solvent system consisted of ultrapure water (A) and acetonitrile (B), both acidified with 0.1% formic acid, with a flow rate of 0.4 mL min^−1^. The sample injection volume was 10 μL.

To ensure accurate mass measurements (i.e., mass deviations ≤ 5 ppm), the instrument was calibrated using ready-to-use calibration solutions (Thermo Fisher Scientific, San José, CA, USA). A mixture of analytical standards was injected at the beginning and end of each sample batch to allow for targeted metabolic profiling (Tier 1) and to evaluate the operational conditions of the instrument (Table S4). Quality control (QC) samples, prepared from pooled biological samples, were injected at the start of the analysis to condition the system and after every ten biological samples to account for instrumental drift.

### Salivary metabolome targeted profiling and untargeted fingerprinting

Targeted HRMS full-scan data processing, including metabolite identification (Tier 1)^53^ was carried out using Xcalibur 4.7 (Thermo Fisher Scientific, San José, CA, USA) for the OPERA cohort and our in-house TARDIS 0.1.0 package (https://github.com/UGent-LIMET/TARDIS) for the other cohorts.^54^ Compound identification was based on the *m/z-*value of the molecular ion, with an allowed mass deviation of ≤ 5 ppm. Using TARDIS, a retention window of ± 9 s was applied, while with Xcalibur a shift of 0.2 min was allowed, based on the retention time of the internal standards. For both TARDIS and Xcalibur peak areas were calculated.^27^ Untargeted full-scan data analysis was conducted using Compound Discoverer™ 3.3 (Thermo Fischer Scientific, San José, CA, USA), enabling simultaneous processing in both positive and negative ion modes. Key parameters for feature extraction included a minimum peak intensity of 500,000 a.u., retention time width of 0.2 min, a mass window of 5 ppm and a peak rating ≥ 4 with default settings in at least 5% of all samples. Detected features were characterized by molecular weight, *m/z*-value, retention time and peak intensity. Missing values in the targeted and untargeted metabolomics data were handled using gap-filling algorithms.^55^, ^56^, ^57^ Targeted metabolites showing QC variability > 20% were excluded.^58^

### V3–V4 16S rRNA gene sequencing for salivary microbiome analysis

Microbiome data for the Fin-HIT and PANIC cohorts were generated at different time points using cohort specific microbiome sequencing and data processing (bioinformatics pipelines and reference databases for taxonomic assignment) protocols (Table S5). To minimise batch effects, we restricted interpretation to phyla consistently detected across both cohorts and performed all microbiome–metabolome correlation analyses within cohorts, comparing results based on directional agreement rather than effect sizes. Details on the 16S rRNA gene sequencing performed on saliva samples are provided elsewhere.^59^, ^60^, ^61^

DNA extraction was performed at the Technology Centre, Sequencing Unit, Institute for Molecular Medicine Finland (FIMM) in 2015 for Fin-HIT and 2022 for PANIC. Extraction included an intensive lysis step using a cocktail of lysozyme with mechanical disruption of the bacterial cells using bead-beating, after which the V3–V4 regions of the 16S rRNA gene were amplified using 16S primers.

For the Fin-HIT cohort the TruSeq (TS)-tailed 1-step amplification protocol was used. The sequencing of PCR amplicons was performed using the 2 × 270 bp sequencing on the Illumina HiSeq1500 instrument (Illumina, Inc., San Diego, CA, USA). Quality filtering was carried out, and sequences were processed using the MiSeq SOP in the mothur pipeline (version 1.35.1).^62^ High-quality assembled reads were aligned to the SILVA 16S rRNA database (version 119),^63^ clustered into operational taxonomic units (OTUs) at a cut-off value > 98% and assigned taxonomy to OTUs using the SILVA bacteria taxonomy.^61^

For the PANIC cohort, sequencing libraries were prepared using the TruSeq (TS)-switched tail protocol. The sequencing of PCR amplicons was performed using the 2 × 301 bp sequencing on the Illumina MiSeq PE300 instrument (Illumina, Inc., San Diego, CA, USA). Quality filtering and sequence processing were carried out using the CLC Genomics Workbench, version 22 (https://digitalinsights.qiagen.com). The SILVA 16S rRNA database (version 132) and taxonomy were used for the alignment and classification of sequences into OTUs with at least 97% similarity.^60^

To improve consistency across cohorts, taxonomic annotations from each database were harmonised by matching names across multiple ranks to their validly published counterparts, as recognized by the International Committee on Systematics of Prokaryotes (ICSP), in accordance with the International Code of Nomenclature of Prokaryotes (ICNP) and the List of Prokaryotic Names with Standing in Nomenclature (LPSN) (Table S6).^64^, ^65^, ^66^

### Statistics

Given the methodological differences across cohorts, including variations in mental wellbeing and dietary intake questionnaires, saliva collection and microbiome analysis protocols, each cohort was analysed independently using a uniform statistical workflow, conducted in R version 4.4.1. This per-cohort analysis served as a sensitivity analysis to evaluate the robustness of the associations observed across populations. More specifically, we aimed to unravel consistent associations that could be replicated across cohorts and as such reduce potential biases introduced by forced scaling or imputation of non-equivalent metadata.

Mental wellbeing was assessed using cohort-specific questionnaires by applying a summary psychological stress *z*-score to facilitate comparability via a measure of general mental wellbeing. Within each cohort the *z*-score-transformed values of all available psychological stress measures were summed to ensure equal weighting of all components. For the FAME cohort, the *z-*score-transformed CDI-2 and PSS were summed, and RSES was subtracted, as higher RSES scores indicate better mental wellbeing. For the Fin-HIT cohort, the absolute value of the *z-*score-transformed body image was used, with the RSES subtracted. In the OPERA cohort, the *z-*score-transformed PSS, CDI-2, and negative affect items from the PANAS-C were summed. For the PANIC cohort, only sleep was considered.

Food consumption levels were categorised based on the available answer options provided in the FFQ for reporting consumption frequency. In the FAME and OPERA cohorts, the options “(almost) never” and “a few times a week” were classified as low consumption, while “(almost) daily” and “multiple times a day” were classified as high consumption. This classification was chosen because the FFQ provided only four answer options, and using a median split would have resulted in unbalanced groups. For the Fin-HIT and PANIC cohorts, high- and low-consumption groups were determined using a median split of the reported consumption frequencies for every food item. To enhance comparability across cohorts, food group variables in FAME, PANIC and OPERA were harmonised by aggregating similar items into broader categories (e.g., combining “poultry with fat” and “poultry without fat” into “poultry”). This harmonisation was not possible for Fin-HIT due to the limited level of detail provided in the used FFQ (Tables S1–S3).

Association studies (correlation analysis and univariate statistics) between metabolome data and potential drivers (i.e., age, anthropometrics, mental wellbeing, lifestyle, dietary habits, and salivary microbiome) were performed on QC-normalised (and log-transformed for targeted metabolomics) peak areas. Microbiome data were centred and log-ratio transformed. False discovery rate correction was performed using the Benjamini-Hochberg procedure, which are reported in the text as *q* values. We considered significant correlations with a *q* ≤ 0.20 in line with earlier work in which this threshold was adopted, particularly in exploratory, high-dimensional metabolomics and microbiome studies.^67^, ^68^, ^69^, ^70^, ^71^

Spearman's correlation (ρ) (stats package (version 3.6.2) and pcor.test from the ppcor R package (version 1.1)) were conducted on both targeted and untargeted metabolomics data as a straightforward, exploratory approach to identify potential associations across a broad range of variables and metabolites. When necessary, i.e., when significant influences from age and/or sex were observed, partial Spearman correlation analyses were used to adjust for these confounders (Table 1). In the FAME cohort, correlations with BF%, waist-to-height (WtoH), PSS, and restrained and emotional eating were corrected for sex, while CDI-2 and RSES were corrected for both sex and age. In Fin-HIT, correlations with WtoH, screen time, and RSES were corrected for sex. In OPERA, correlations with BF%, PSS, and hair cortisol were corrected for sex, while correlations with PANAS-C were corrected for both age and sex. In PANIC, correlations with BF%, PA, and screen time were corrected for sex. Functional analysis was achieved with MetaboAnalyst 6.0, using the Mummichog algorithm and the MFN Python package, by ranking the negatively ionized *m/z*-values per parameter by ascending *p*-values. Metabolic overrepresentation was considered significant with a *p*(γ) ≤ 0.10 to indicate pathways with a greater functional relevance.^72^

Pairwise univariate comparisons were performed using the two-sided Welch's t-test for normally distributed data (Shapiro–Wilk *p* > 0.05 for all groups) or the two-sided Wilcoxon rank-sum test for non-normally distributed data (Shapiro–Wilk *p* ≤ 0.05 for at least one group). For multiple comparisons, either the one-way ANOVA (Shapiro–Wilk *p* > 0.05) or the two-sided Kruskal–Wallis rank-sum test (Shapiro–Wilk *p* ≤ 0.05) was used, followed by post hoc tests (Tukey or Dunn following ANOVA or Kruskal–Wallis). Participants were grouped according to whether they were of a healthy weight or overweight (incl. obese), while mental wellbeing was categorised using the 50% lowest and highest psychological stress *z*-scores. Multiple comparisons for weight categories included healthy weight, overweight and obesity, classified based on BMI*z*.^30^ For mental wellbeing, groups were divided into low, moderate and high psychological stress, defined by the 25% lowest and highest psychological stress *z*-scores. Correction for age and/or sex as confounding factors was performed for normally distributed data (Table 1). For non-normally distributed data, confounders were not considered, as this is not possible using standard rank-based tests.^73^ In the FAME cohort, mental wellbeing was corrected for age and sex, while weight was corrected for age in FAME and PANIC. In Fin-HIT, OPERA, and PANIC, mental wellbeing was corrected for sex. Categorical data were analysed via the chi-squared test using the chisq.test in the stats package (version 3.6.2) in R.

### Ethics

Recruitment across all cohorts was conducted in accordance with the Declaration of Helsinki, with approval from the relevant institutional review boards. The FAME study was approved by the central Ethics Committee at Ghent University Hospital (BC-10583) as well as by the Ethics Committees of University Hospital Antwerp and Ghent General Hospital Jan Palfijn. The Fin-HIT study was approved by the Coordinating Ethics Committee of the Hospital District of Helsinki and Uusimaa, Finland (169/13/03/00/10). The PANIC study was approved by the Research Ethics Committee of the Hospital District of Northern Savo, Finland (69/2006). The OPERA study was approved by the Ethics Committee of Ghent University Hospital, Belgium (EC2017/0527). Written informed consent was obtained from all participants, including minors, or from their legal guardians where applicable.

### Role of funders

The funders of this study did not participate in the design, data collection, analysis, interpretation, or writing of this report. The decision to submit the manuscript for publication was made solely by the authors listed, without any influence from the funders.

## Results

### Paediatric cohort characteristics

In this cross-sectional study, 1436 children and adolescents from two Belgian (FAME and OPERA) and two Finnish (PANIC and Fin-HIT) cohorts delivered one saliva sample at one timepoint each. Data on age, anthropometrics, mental wellbeing, lifestyle, dietary intake and microbiome composition were collected as well (Fig. 1).

FAME is an observational study focusing on the links between dietary habits, psychological stress and metabolic health in Belgian adolescents.^31^ Fin-HIT is a study that aims to explore environmental and genetic determinants of body weight and weight gain in children followed up until adolescence and finally adulthood, as well as the aetiology and molecular mechanisms underlying obesity and related health outcomes.^32^ PANIC is an 8-year controlled PA and dietary intervention study in a population sample of Finnish children followed up until adolescence.^33^^,^^34^ OPERA focuses on the role of emotion regulation within the chronic stress–obesity axis of a sample of Flemish adolescents.^35^

In the FAME, Fin-HIT, OPERA and PANIC cohorts, 14.5% and 10.6%, 40.0% and 10.7%, 21.3% and 10.3% and 9.9% and 2.4% of the included participants had overweight or obesity, respectively. Several differences in participants’ characteristics according to age and sex were also detected across cohorts. Girls generally exhibited higher BF% and lower WtoH-ratios (*p* ≤ 0.05), except in the validation cohorts, where no difference in WtoH was observed. In terms of mental wellbeing, girls scored generally worse, with higher psychological stress *z*-scores (*p* ≤ 0.05) and higher reported levels on perceived stress (PSS), depressive feelings (CDI-2), negative feelings (PANAS-C), and measured hair cortisol, along with more restrained and emotional eating behaviour and lower self-esteem (RSES). Boys, on the other hand, reported more screen time (*p* < 0.001) in Fin-HIT, but less in PANIC (*p* < 0.001), and demonstrated higher levels of PA in Fin-HIT and PANIC (*p* ≤ 0.05). In the FAME cohort, age was positively correlated (*p* ≤ 0.05) with deteriorating mental wellbeing measures, such as CDI-2 and RSES, while negative feelings (PANAS-C) increased with age in OPERA (Table 1).

Differences by weight status as categorised according to Cole et al.^30^ were observed in all cohorts. Additionally, participants with overweight and obesity reported inferior mental wellbeing, such as more perceived stress (PSS), depressive feelings (CDI-2), restrained, emotional, and external eating behaviour in the FAME cohort, and a lower self-esteem (RSES) in both FAME and Fin-HIT, in addition to a more disturbed body image in Fin-HIT. In OPERA, higher scores were observed for external eating and depressive feelings (CDI-2) in participants with overweight and obesity. Overall, these individuals noted higher psychological stress *z*-scores and more screen time than their peers with a healthy weight (*p* ≤ 0.05). No differences (*p* > 0.05) were observed in PA or sleep duration in the Fin-HIT, PANIC and OPERA cohorts. In the FAME cohort, participants with overweight and obesity were slightly older, while in the PANIC cohort, they tended to be younger. No sex differences (*p* > 0.05) were observed across the different weight categories for the four cohorts combined (Table 1).

Salivary metabolites from all participants were analysed using our in-house optimised and validated UHPLC-Q-Orbitrap-HRMS methodology.^27^ Data were processed in an untargeted fashion in the exploratory FAME and Fin-HIT cohorts, yielding respectively 8317 and 3598 metabolic features. Next, a total of 302 metabolites, representing multiple physicochemical classes^74^ were assessed in a targeted manner in the FAME cohort. Of these, 188 were consistently detected with a CV < 20% in the QCs of FAME and/or Fin-HIT and further processed in the validation cohorts if relevant (Table S4).

### Correlations between children and adolescents' salivary metabolome and their age, anthropometrics, mental wellbeing and lifestyle

**Fig. 2:**
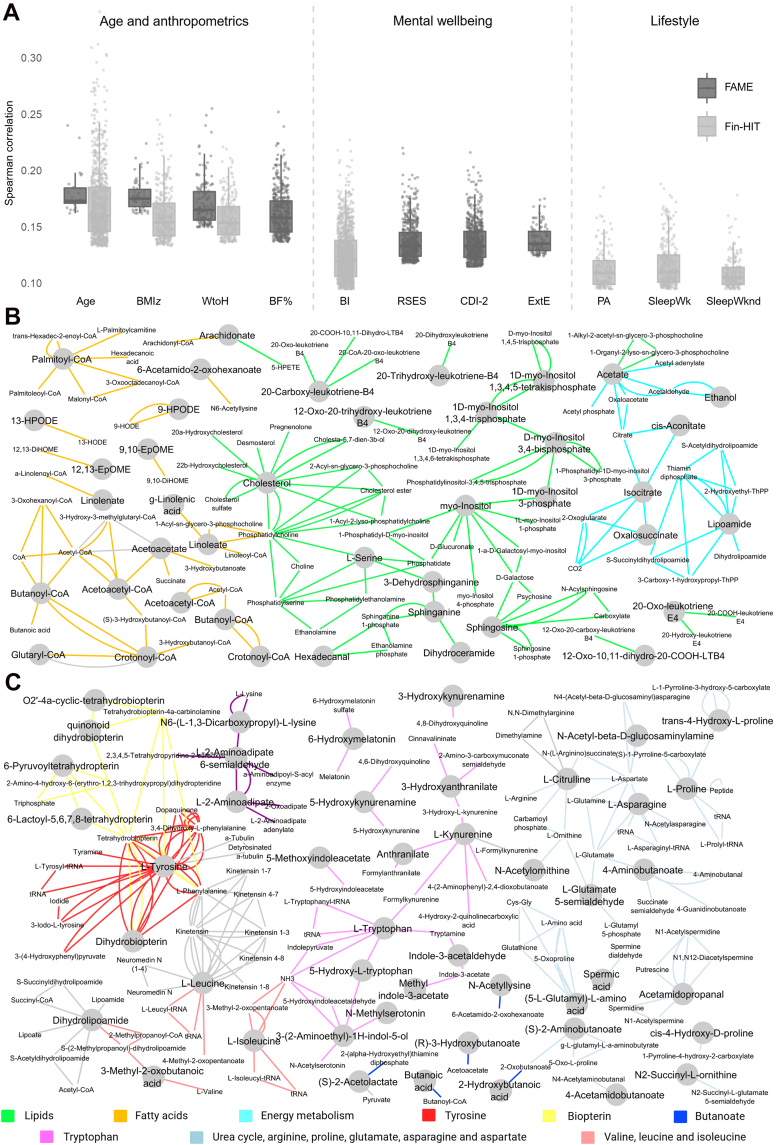
Paediatric salivary metabolome correlations and functional analysis with age, anthropometrics, mental wellbeing and lifestyle in the exploratory FAME and Fin-HIT cohorts. (**A**) Spearman correlations (*q* ≤ 0.05) of salivary metabolites with age, BMI*z*, body fat percentage (BF%) (only in FAME), and waist-to-height (WtoH). Spearman correlations (*q* ≤ 0.20) of salivary metabolites with mental wellbeing: body image (BI) in Fin-HIT, Rosenberg Self-Esteem Scale (RSES) in Fin-HIT and FAME, although no significant correlations could be retained for Fin-HIT, Children's Depression Inventory-2 (CDI-2) and external eating (ExtE) in FAME and lifestyle (physical activity (PA), sleep duration on weekdays (SleepWk) and sleep duration on weekends (SleepWknd)) in Fin-HIT. (**B**) Metabolic pathways (*p*(γ) ≤ 0.10) overrepresented in the salivary metabolome (Fin-HIT cohort) correlated with age. (**C**) Metabolic pathways (*p*(γ) ≤ 0.10) overrepresented in the salivary metabolome (FAME cohort) correlated with BF%. Grey circles = imported data, no circles = related metabolites (created with MetScape).

We evaluated the impact of age, anthropometrics, mental wellbeing and lifestyle on the untargeted salivary metabolome using (partial) Spearman correlation analysis in the exploratory FAME and Fin-HIT cohorts (Fig. 2A).

In the FAME cohort, the strongest metabolome correlations (*q* ≤ 0.05) were observed with anthropometric measures, including BF% (ρ |0.137–0.252|, 431 features), WtoH (ρ |0.150–0.255|, 152 features) and BMI*z* (ρ |0.162–0.224|, 62 features), and age (ρ |0.162–0.240|, 27 features). In the Fin-HIT cohort, the highest correlations were found with age (ρ |0.133–0.341|, 835 features), BMI*z* (ρ |0.133–0.249|, 289 features) and WtoH (ρ |0.136–0.219|, 222 features).

Functional analysis (*p*(γ) ≤ 0.10) of age-correlated salivary metabolites in the FAME cohort indicated altered amino acid, nucleotide (pyrimidine and purine), butanoate, and nitrogen pathways (Table S8). Similarly, in Fin-HIT, age-correlated salivary metabolites comprised amino acids, butanoate and pyrimidine, along with pathways related to lipid, fatty acid, vitamins (B6 and biotin), and energy metabolism (carnitine shuttle, TCA cycle) (Fig. 2B, Table S8). WtoH-correlated salivary metabolites in FAME comprised purine, amino acids, butanoate, pyruvate, nitrogen, and vitamin B3 metabolism (Table S9). In Fin-HIT, similarly, pathways involved amino acids and butanoate, along with fatty acid, linoleate, vitamin E, glycerophospholipid and glycosphingolipid metabolism (Table S10). The metabolic pathways associated with BMI*z* in FAME included amino acids, purine, butanoate, vitamin B3 and nitrogen metabolism (Table S11), alongside fatty acid, energy and vitamin metabolism in Fin-HIT (Table S12). Salivary metabolites correlating well with BF% in FAME were involved in amino acid and butanoate metabolism (Fig. 2C, Table S13).

With regard to mental wellbeing, 2 metabolites correlated (*q* ≤ 0.20) with self-esteem (RSES), 13 with depressive feelings (CDI-2), and 2 with external eating in the FAME cohort. In the Fin-HIT cohort, none of the correlations with self-esteem (RSES) were significant, but 335 correlations were identified with BI (ρ |0.133–0.227|). Functional analysis (*p*(γ) ≤ 0.10) revealed that several amino acid pathways, including β-alanine, glycine, serine, alanine, threonine, aspartate, asparagine, arginine and proline were associated with depressive feelings (CDI-2) in FAME (Table S14). Moreover, perceived stress (PSS) could be linked to energy (i.e., carbon fixation, glycolysis, gluconeogenesis, fatty acids and tricarboxylic acid cycle) and nucleotide (pyrimidine and purine) metabolism (Table S15), while self-esteem (RSES) was associated exclusively with arginine and proline metabolism (Table S16). In the Fin-HIT cohort, self-esteem (RSES) revealed correlations with altered fatty acid, amino acid, and energy pathways, including arginine and proline metabolism (Table S17). Pathways related to emotional, external, and restrained eating in FAME were predominantly tied to amino acids (Tables S18–S20).

Lifestyle factors (PA, screen time, sleep duration) were only assessed in the exploratory Fin-HIT cohort. The salivary metabolome showed stronger correlations (*q* ≤ 0.20) with PA (ρ |0.134–0.185|, 19 features) compared to screen time, for which no significant correlations were observed. Sleep duration correlations were higher during the week (ρ |0.136–0.188|, 45 features) than during the weekend (ρ |0.136–0.178|, 8 features).

### Metabolic indicators of overweight and obesity detected in saliva across all cohorts

Targeted salivary metabolome analysis identified a total of 93 and 9 metabolites that correlated with key measures such as BMI*z*, BF%, and WtoH ratio in the FAME and Fin-HIT cohorts, respectively. Of these, 6 metabolites correlated with these measures in both cohorts: N6-acetyllysine and N-acetylmethionine with WtoH, putrescine, N-acetylvaline and 3-indoleacetic acid with BMI*z*, l-glutamic acid with both BMI*z* and WtoH (Table S21). Pairwise and multiple univariate analysis of these 96 correlated metabolites revealed different (*q* ≤ 0.20) QC-normalised, log-transformed median peak areas for, respectively, 91 and 74 metabolites in FAME and 4 and 16 metabolites in Fin-HIT, in participants with overweight and obesity (Tables S22–25).

**Fig. 3:**
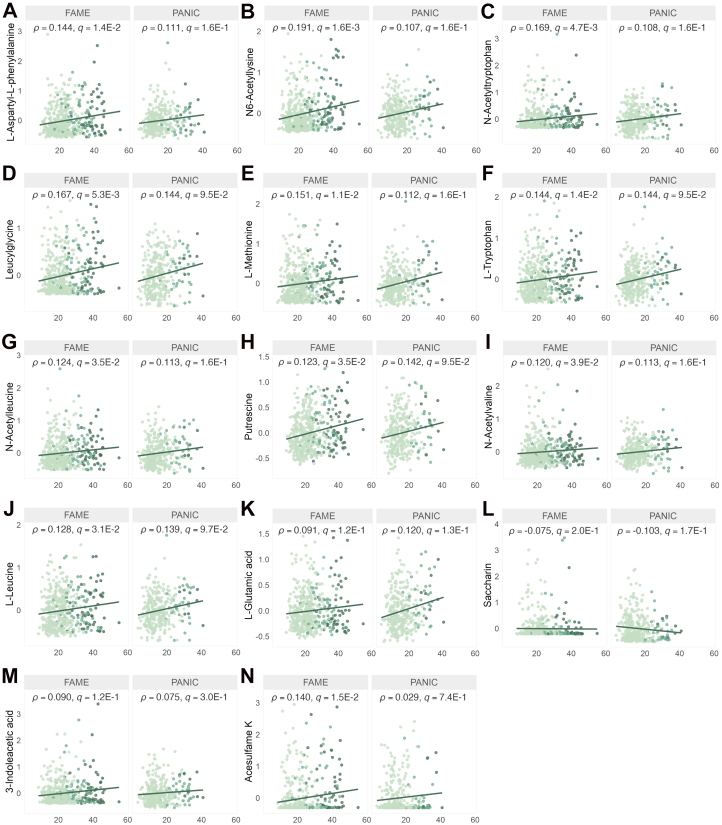
Correlation plots of body fat percentage (BF%) and salivary metabolites in the exploratory FAME and validation PANIC cohorts. The y-axis represents QC-normalised, log-transformed, Pareto-scaled peak areas of salivary metabolites; the x-axis represents BF%. Shown metabolites include: (**A**) L-aspartyl-l-phenylalanine, (**B**) N6-acetyllysine, (**C**) N-acetyltryptophan, (**D**) leucylglycine, (**E**) l-methionine, (**F**) l-tryptophan, (**G**) N-acetylleucine, (**H**) putrescine, (**I**) N-acetylvaline, (**J**) l-leucine, (**K**) l-glutamic acid, (**L**) saccharin, (**M**) 3-indoleacetic acid and (**N**) acesulfame K. Colouring of the dots according to BMI*z*: healthy weight (light green), overweight (green), obesity (dark green).^30^

The association (significant correlation and/or univariate differences (*q* ≤ 0.20)) of 13 metabolites with weight status could be consistently detected in at least three cohorts, i.e., both exploratory and validation, of which the correlations with BF% in the exploratory FAME and validation PANIC cohort are presented in Fig. 3. Multiple other metabolites were confirmed by correlation analysis and/or univariate comparison in at least one validation cohort, predominantly comprising amino acids and their derivatives, and nucleotides. Acesulfame K (AceK) was consistently correlated with BF% and WtoH in both Belgian cohorts (Tables S21–S27).

### Salivary metabolites related to mental wellbeing and lifestyle across cohorts

**Fig. 4:**
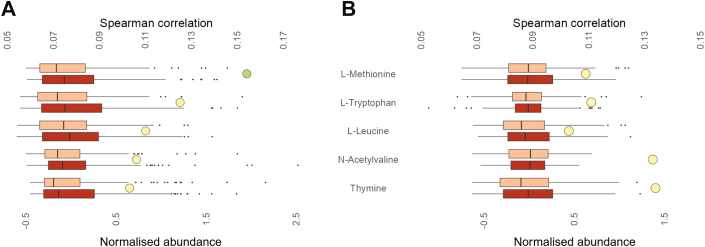
Metabolic alterations consistently associated with mental wellbeing in both validation cohorts, in children and adolescents. Boxplots of the pairwise comparisons of QC-normalised, log-transformed, and Pareto-scaled peak areas, consistently detected in both exploratory cohorts, between low (light orange) and high psychological stress *z*-score (dark orange). Metabolites' correlation with mental wellbeing parameters for (**A**) FAME and (**B**) Fin-HIT are marked with a circle. Green circle: significant correlation (*q* ≤ 0.05); yellow circle: significant correlation (0.05 < *q* ≤ 0.20).

Mental wellbeing questionnaire data, i.e. depressive feelings (CDI-2), perceived stress (PSS) and external eating correlated (*q* ≤ 0.20) with 35 metabolites in the FAME cohort. Although included as a lifestyle parameter, sleep duration was also regarded as a proxy for mental wellbeing in the Fin-HIT and PANIC cohorts. In the Fin-HIT cohort, 11 metabolites correlated with body image, sleep duration on weekdays and weekends and self-esteem (RSES), of which 5 overlapped with the FAME cohort, i.e., l-methionine, l-tryptophan, l-leucine, thymine and N-acetylvaline (Table S28). Data of these 5 metabolites, consistently detected and associated with mental wellbeing in both exploratory cohorts are presented in Fig. 4. Of these, the (acetylated) amino acids, but not thymine, were also consistently associated (significant correlation and/or univariate differences (*q* ≤ 0.20)) with weight status in at least three cohorts (Table S21). Of the 41 metabolites that correlated with mental wellbeing in FAME and/or Fin-HIT, pairwise and multiple univariate analysis revealed significantly (*q* ≤ 0.20) different QC-normalised, log-transformed median peak areas for 4 metabolites in FAME in participants with moderate and high levels of psychological stress (Table S28). The association (significant correlation and/or univariate differences (*q* ≤ 0.20)) of l-methionine, l-tryptophan, l-leucine and thymine with mental wellbeing could be consistently observed in three cohorts, while multiple other metabolites were confirmed by correlation analysis and/or univariate comparison in at least two cohorts, again predominantly comprising amino acids and their derivatives, and nucleotides. The association of AceK with low mental wellbeing was confirmed in both Belgian cohorts (Tables S28–S32).

In the Fin-HIT cohort, the lifestyle factor screen time displayed no significant correlations (*q* ≤ 0.20) with any of the targeted metabolites, whereas PA showed a significant (*q* ≤ 0.20) positive correlation with l-glutamic acid and negative correlation with N-acetylleucine (Table S33). Notably, while N-acetylleucine was also significantly correlated with PA in the PANIC cohort, the direction of correlation differed (Table S34).

### Artificial sweeteners consistently detected in paediatric saliva and their association with food intake, weight status and mental wellbeing

Targeted analysis revealed a total of 86 correlations (*q* ≤ 0.20) between the intake of specific food items and 61 and 5 metabolites in the exploratory FAME and Fin-HIT cohorts, respectively (Tables S35 and 36). Significant positive correlations (*q* ≤ 0.05) were observed between the consumption of diet carbonated drinks and both AceK and L-aspartyl-l-phenylalanine, a metabolite produced through the hydrolysis of aspartame, in the FAME cohort, while positive correlations (*q* ≤ 0.20) were also detected between AceK and the intake of soft drinks (incl. sugared and diet) in Fin-HIT and savoury snacks in the FAME cohort (Tables S35–S36). Pairwise univariate analysis of these 66 correlated metabolites identified different (*q* ≤ 0.20) QC-normalised, log-transformed median peak areas for 63 metabolites in FAME and 13 in Fin-HIT when comparing children and adolescents reporting low vs. high consumption of specific food items. More specifically, 3-indoleacetic acid, thymine and hypoxanthine were lower, while theobromine was higher upon high consumption of chocolate-based food items in both exploratory cohorts. In addition, caffeine and AceK were positively associated with the consumption of soft drinks in both cohorts (Tables S36–S38).

**Fig. 5:**
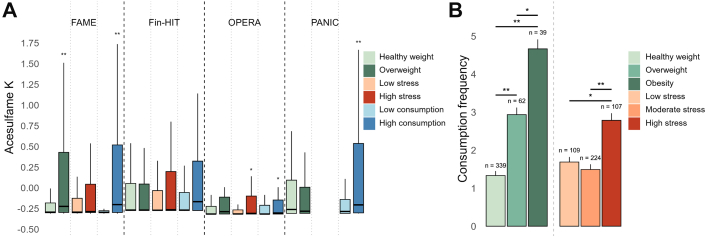
Detection of acesulfame K (AceK) in children and adolescents' saliva across cohorts, and its associations with weight status, mental wellbeing and the intake of specific food items. (**A**) Pairwise univariate comparisons of QC-normalised, log-transformed and Pareto-scaled AceK levels (Y-axis) between participants with healthy weight and overweight (incl. obese) based on BMI*z*,^30^ low and high stress based on psychological stress *z-*score, and low and high consumption groups of the most strongly correlating food items. In the FAME cohort, the food item was carbonated diet drinks; in Fin-HIT, soft drinks (incl. sugared and diet), in PANIC, artificially sweetened beverages, and in OPERA, frozen snacks. (**B**) Multiple univariate comparisons of the weekly consumption frequency (mean ± standard error) of diet carbonated drinks by weight status and psychological stress level in the FAME cohort. ∗∗*q* ≤ 0.05, ∗*q* ≤ 0.20.

Higher levels of AceK were observed with high consumption (*q* ≤ 0.20) of, respectively, artificially sweetened beverages and sweetened yogurt and frozen snacks in the validation cohorts PANIC and OPERA (Fig. 5A, Tables S39–S40). Interestingly, levels of AceK were also correlated with depressive feelings within the FAME (*q* ≤ 0.05) cohort, a finding further corroborated by increased levels of AceK in the highly psychologically stressed group in the OPERA cohort (*q* ≤ 0.20; Fig. 5A, Tables S28, S31, S32). Even more, multiple univariate comparisons revealed that the consumption of carbonated diet drinks and savoury snacks was significantly higher (*q* ≤ 0.05) in participants with obesity compared to their peers with a healthy weight or overweight, and in those with high vs. moderate psychological stress in FAME (Fig. 5B, Tables S42 and 43). Similarly, consumption of soft drinks in Fin-HIT, artificially sweetened beverages in PANIC, and diet drinks and frozen snacks in OPERA was higher among participants with obesity and/or overweight (Tables S44–S46). Increased consumption of diet drinks was also associated with high psychological stress levels in Fin-HIT, but not in OPERA and PANIC (Tables S47–S49).

### Correlation of consistently detected weight- and mental wellbeing-related salivary metabolites with their microbial composition

We assessed the impact of the microbiome on the salivary metabolome using Spearman correlation analysis in the exploratory Fin-HIT and validation PANIC cohort. Targeted analysis revealed a total of 1314 correlations (*q* ≤ 0.20) between the relative abundance of 9 phyla, consisting of 80 bacteria (genus level), and 29 metabolites in the Fin-HIT cohort (Table S50). These included all 13 metabolites that were consistently associated (correlation and/or univariate differences (*q* ≤ 0.20)) with weight in at least 3 cohorts (Table S21). Of these, 4 metabolites, along with thymine, were also associated with mental wellbeing in both exploratory cohorts (Table S28). All weight-related metabolites and thymine showed significant (*q* ≤ 0.20) and predominantly positive correlations with multiple genera within the Bacteroidota, Bacillota, Actinomycetota and Pseudomonadota phyla. For AceK, a positive correlation (*q* ≤ 0.20) was noted with the *Lactococcus* genus, belonging to the Bacillota phylum (Table S50).

Building on the findings from the Fin-HIT cohort, we again observed in the PANIC cohort that all 13 weight-related metabolites and thymine correlated (*q* ≤ 0.20) with microbial phyla such as Bacteroidota, Pseudomonadota, Actinomycetota and Bacillota, but less with Fusobacteriota (Table S51). At the genus level, several correlations from the Fin-HIT cohort were replicated in PANIC. In this regard, the majority of the weight-related metabolites correlated positively with *Actinomyces*, *Prevotella*, *Veillonella*, *Oribacterium* and *Rothia*. For N-acetyltryptophan, negative correlations with *Streptococcus* were noted. l-Methionine and l-leucine correlated positively with *Campylobacter*, while putrescine and 3-indoleacetic acid correlated negatively with *Corynebacterium*. Additionally, 3-indoleacetic acid correlated positively with *Megasphaera* but negatively with *Capnocytophaga*. Finally, saccharin correlated negatively with *Capnocytophaga*, *Granulicatella*, *Haemophilus*, *Neisseria*, *Porphyromonas* and *Streptobacillus*.

## Discussion

By generating large-scale salivary metabolomics data from 1436 children and adolescents, we explored to what extent the salivary metabolome reflects age, anthropometrics, mental wellbeing, lifestyle, dietary intake, and microbial composition in two European countries (Belgium and Finland). For this purpose, we utilised our unique fully validated UHPLC-HRMS-based metabolic profiling and fingerprinting method.^27^ Saliva is an emerging biofluid in epidemiology and diagnostics, offering multiple advantages over more widely used biofluids such as blood and urine, particularly in children.^26^ Indeed, its intra-individual variability has been shown to be modest and often lower than that of urine or even plasma.^75^, ^76^, ^77^ However, to fully harness the potential of saliva, an assessment of its physiological and external modifiers is crucial.^22^^,^^23^ Although paediatric salivary metabolome data have been collected in some previous studies, their sample sizes were either very small (n < 100) or the analytical methods applied not specifically optimised (nor validated) towards saliva.^19^, ^20^, ^21^ Furthermore, the majority of metabolomics studies to date has been performed in single cohorts, which constrains the generalisability and cross-cohort validation of suggested biomarkers.^22^^,^^23^ To the best of our knowledge, our study is the first to present both untargeted and targeted salivary metabolomics data from a large number of children and adolescents, including external validation, while assessing the impact of a broad range of internal and external modifiers. As such, it offers a valuable repository for the paediatric salivary molecular composition and could serve as guidance for exploring its association with children and adolescents’ health.

The correlations observed in this study (ρ |0.133–0.252|) are comparable to those reported in blood-based metabolomics studies, which have generally been modest, with ρ values ranging from approximately |0.12| to |0.35|, thereby reinforcing saliva's potential to yield biologically meaningful results.^67^^,^^71^^,^^78^^,^^79^ In our exploratory cohorts (FAME and Fin-HIT), the individual salivary metabolic fingerprint showed the strongest correlations with weight status and age, although the age range of the cohorts determined the parameter with the highest overall impact. Indeed, in the older FAME participants, weight status dominated the correlation matrix, while age defined the metabolic fingerprint of the younger and more narrow age-ranged Fin-HIT participants better. We further demonstrated that multiple targeted salivary metabolites, significantly correlated with weight status and mental wellbeing, were consistently detected across cohorts, despite variability in sample collection methods and key metadata assessment. Indeed, variation in saliva collection across cohorts may have affected the salivary metabolome. More specifically, studies have shown that factors like the use of stimulants (e.g., paraffin in PANIC), the collection method (active or passive) and time (morning vs. afternoon), pre-collection procedures (e.g., fasting status), and storage conditions can alter metabolite concentrations and profiles.^80^, ^81^, ^82^ Furthermore, microbiome sequencing and data processing (bioinformatics pipelines and reference databases for taxonomic assignment) varied across cohorts. Therefore, we did not evaluate microbial community composition directly,^83^ but focused on within-cohort correlations between microbial taxa and salivary metabolites and assessed the directional consistency of these associations across cohorts. This strategy minimised sensitivity to technical variation in absolute abundances and offered a pragmatic evaluation of the robustness of associations under real-world conditions. Next, mental wellbeing measures were highly variable between cohorts, i.e., CDI-2, RSES, PSS, eating behaviour in FAME, body image, RSES, sleep duration on weekdays and weekends in Fin-HIT, CDI-2, eating behaviour, PSS, hair cortisol and PANAS-C in OPERA, and sleep duration in PANIC. Finally, dietary intake was assessed via different FFQs or food records, with differences in item formulation, recall periods, and cultural context across countries. Moreover, the distributions of the consumption of most food items were rather unbalanced, regardless of cohort and country. Nevertheless, several intact and acetylated amino acids were consistently increased in the saliva of participants with overweight and obesity as well as in children and adolescents with high levels of psychological stress, which may reflect the co-occurrence of overweight and a lower mental wellbeing, suggesting shared biological underpinnings that warrant further investigation.^16^ Elevated plasma levels of the intact amino acid leucine have been widely observed in young individuals with overweight and obesity, while increases in methionine and tryptophan have been reported in blood as well.^7^ The increased detection of multiple acetylated (and methylated) amino acids on its turn may be reflective of an enhanced histone acetylation, resulting from epigenetic modifications, as recently evidenced in a study on blood samples from children with obesity.^84^ Putrescine, a polyamine derived from arginine that has been detected as the most abundant polyamine in human saliva,^85^ was also reproducibly detected in our study and correlated positively with weight status. Our analysis of the salivary microbiome further revealed associations between putrescine and several bacterial taxa, aligning with previous findings that elevated putrescine levels associate with alterations in the oral microbiome.^86^ Indeed, disturbance of the oral microbiome composition between weight categories in children was demonstrated in a review by de Lemos et al., where nine out of 11 studies demonstrated that microbial signatures in the oral cavity to a great extent parallel obesity-associated gut microbiota.^87^ Interestingly, AceK, a synthetic sugar substitute, was consistently detected in all four cohorts irrespective of the sampling protocol followed, and to the best of our knowledge for the first time in saliva of such a large group of children and adolescents. Moreover, salivary AceK was positively associated with weight status and psychological stress in both Belgian cohorts and the consumption of foods known to contain AceK in all cohorts. The former association may be explained by the worldwide rise in artificial sweetener consumption over the past decade,^88^, ^89^, ^90^, ^91^, ^92^ the higher intake of soft drinks among older children,^93^^,^^94^ or the generally higher soft drink consumption in Belgium compared to Finland.^93^ The latter on its turn showed to be higher in participants with overweight and obesity and in those reporting increased psychological stress. Finally, in the Fin-HIT cohort, a positive association between AceK and the relative abundance of Bacillota was observed. Indeed, AceK consumption has been shown to perturb the gut microbiome of CD-1 mice and C57BL/6 mice.^95^^,^^96^ The observed weight gain, the shifts in gut bacterial community composition, and the enrichment of functional bacterial genes related to energy metabolism were however highly sex-specific in the CD-1 mice. Lin et al. found that AceK consumption enhanced hepatic lipogenesis and decreased β-oxidation in ApoE^−/−^ mice. In addition, it directly increased lipogenesis and decreased β-oxidation in HepG2 cells. When exposing pregnant and lactating mice to AceK at doses relevant for human consumption, metabolic changes in the pups were drastic, indicating extensive downregulation of hepatic detoxification mechanisms and changes in bacterial metabolites. Microbiome profiling confirmed a significant increase in Bacillota and a striking decrease of *Akkermansia muciniphila*.^97^ Similar microbiome alterations have been reported for obesity and metabolic disease in adults^98^^,^^99^ and thus support our findings in children and adolescents. Moreover, studies with mice have also demonstrated that long-term AceK intake in combination with a low carbohydrate diet affects the cognitive function through the reduction of cortical glucose levels.^100^ However, findings on AceK intake in relation to weight status, mental wellbeing or overall health status in humans are either underpowered or inconclusive because of complex background diets.^101^ The positive associations of weight category and psychological stress with salivary AceK in our Belgian cohorts prompt further mechanistic research in this area, in which salivary metabolomics may play an important role.

Our study has several strengths. First, the study has a large sample size with age, anthropometric, mental wellbeing, lifestyle, and food consumption data collected simultaneously within all four cohorts across two countries. Second, by integrating salivary metabolomics with microbiome analyses in two cohorts, we offer an additional mechanistic insight into the origin of specific weight-related salivary metabolites. Third, we utilised an analytical method that was specifically optimised towards maximal salivary metabolome coverage in terms of physicochemical diversity of molecules and reproducibility within- and between-batch and enabled both untargeted fingerprinting (> 3500 features) and targeted profiling (302 in-house standards). Fourth, we identified multiple metabolite associations that could be replicated across independent cohorts, despite differences in saliva collection protocols and metadata measurements. While this methodological heterogeneity was challenging, it also provided an opportunity to conduct a sensitivity analysis to assess the robustness of our findings in the face of real-world variability. This underscores the biological relevance of the replicated signals. Finally, the generalisability of our findings is supported by the diversity of the included cohorts, which represent a mix of population-based and targeted sampling strategies. Although the overrepresentation of participants with overweight and obesity in some cohorts may affect the estimation of population-level prevalence, the consistency of associations observed across cohorts with differing compositions supports the robustness and broader applicability of our findings.

There are also several limitations. First, associations from this cross-sectional study are not necessarily causal. Capturing these associations is just a first step in epidemiological research that should provide guidance towards downstream mechanistic investigations as to which physiological processes affect the weight status and how mental wellbeing, lifestyle, food consumption and microbiome composition play a part in that. Second, both validation cohorts, i.e., OPERA and PANIC, together included only 41 participants with overweight and obesity, and in OPERA only 33 participants were classified as having low mental wellbeing, while for PANIC, only sleep was included as mental wellbeing measure. The smaller sample sizes in these subgroups of the validation cohorts may have underpowered the statistical analysis, thereby restricting the ability to validate findings from the exploratory cohorts. Larger replication cohorts for which similarly high-end data on the salivary metabolome, mental wellbeing, lifestyle, diet, and the microbiome are needed to confirm our findings. Moreover, given the limitations of dietary data from FFQs, future studies would benefit from higher resolution dietary assessment methodologies, such as weighed food records.

In conclusion, our results demonstrate that age and weight status correlate best with the salivary metabolome, with multiple metabolites consistently affected by weight and/or mental wellbeing across cohorts. Many associations found here replicate previously reported findings on metabolites detected mainly from blood, supporting the validity of our results. The majority of the findings, however, are novel, making them a useful resource for future research, either for improving molecular understanding of health and disease, or for forming the basis of interventional studies aimed at altering the levels of salivary metabolites.

## Contributors

E.D. and E.C. have contributed equally to the work.

Conceptualisation: E.D.P., E.C., L.V.; Data curation/Resources: E.D.P., E.C., K.W., K.D.W., H.V., M.H.L., T.A.L., K.V.D.M., N.B., R.D.B., S.L., L.V.; Formal analysis: E.D.P., E.C., V.P., M.V.; Methodology: E.D.P., E.C., B.P., K.D.W.; Investigation: E.D.P., E.C., B.P.; Software: M.V., P.V.; Supervision: L.V., S.D.H., N.M., H.V., T.A.L., A.G.; Visualisation: E.D.P., E.C., V.P., M.V.; Writing – original draft: E.D.P., E.C., L.V. ; Writing – review & editing: E.D.P., E.C., K.W., K.D.W., V.P., B.P., P.V., S.D.H., N.M., H.V., M.H.L., T.A.L., K.V.D.M., N.B., R.D.B., S.L., A.G., M.V., L.V.; Funding acquisition: L.V., S.D.H., N.M., H.V., T.A.L.

All authors read and approved the final manuscript. Verification of all underlying data was performed by E.D.P., E.C., M.V. and L.V.

## Data sharing Statement

The metadata and the.mzXML metabolomics files generated in this study are available at the Metabolomics Workbench (https://www.metabolomicsworkbench.org) under Project ID PR002287 (https://doi.org/10.21228/M80Z5F), with study IDs ST003709, ST003706, ST003687, and ST003697 corresponding to PANIC, OPERA, Fin-HIT, and FAME, respectively. All code used in this study is publicly available on GitHub at (https://github.com/UGent-LIMET). This repository contains all scripts and resources necessary to reproduce the analyses and results presented in the manuscript. Detailed instructions for installation and usage are provided in the repository's README file. All data needed to evaluate the conclusions in the paper are present in the paper and/or the Supplementary Materials.

## Declaration of interests

The authors declare that they have no competing interests.

## References

[1] Chen L., Zhernakova D.V., Kurilshikov A. (2022). Influence of the microbiome, diet and genetics on inter-individual variation in the human plasma metabolome. Nat Med.

[2] Pero-Gascon R., Hemeryck L.Y., Poma G. (2022). FLEXiGUT: rationale for exposomics associations with chronic low-grade gut inflammation. Environ Int.

[3] Zeevi D., Korem T., Zmora N. (2015). Personalized nutrition by prediction of glycemic responses. Cell.

[4] Palmnäs M., Brunius C., Shi L. (2020). Perspective: metabotyping - a potential personalized nutrition strategy for precision prevention of cardiometabolic disease. Adv Nutr.

[5] De Spiegeleer M., Plekhova V., Geltmeyer J. (2023). Point-of-care applicable metabotyping using biofluidspecific electrospun MetaSAMPs directly amenable to ambient LA-REIMS. Sci Adv.

[6] Liu N., Xiao J., Gijavanekar C. (2021). Comparison of untargeted metabolomic profiling vs traditional metabolic screening to identify inborn errors of metabolism. JAMA Netw Open.

[7] De Spiegeleer M., De Paepe E., Van Meulebroek L., Gies I., De Schepper J., Vanhaecke L. (2021). Paediatric obesity: a systematic review and pathway mapping of metabolic alterations underlying early disease processes. Mol Med.

[8] Valles-Colomer M., Falony G., Darzi Y. (2019). The neuroactive potential of the human gut microbiota in quality of life and depression. Nat Microbiol.

[9] Pu J., Liu Y., Zhang H. (2021). An integrated meta-analysis of peripheral blood metabolites and biological functions in major depressive disorder. Mol Psychiatry.

[10] Zaghlool S.B., Halama A., Stephan N. (2022). Metabolic and proteomic signatures of type 2 diabetes subtypes in an Arab population. Nat Commun.

[11] Müller J., Bertsch T., Volke J. (2021). Narrative review of metabolomics in cardiovascular disease. J Thorac Dis.

[12] Schmidt D.R., Patel R., Kirsch D.G., Lewis C.A., Vander Heiden M.G., Locasale J.W. (2021). Metabolomics in cancer research and emerging applications in clinical oncology. CA Cancer J Clin.

[13] World Health Organization (2024). Noncommunicable diseases. https://www.who.int/news-room/fact-sheets/detail/noncommunicable-diseases.

[14] Ng M., Fleming T., Robinson M. (2014). Global, regional, and national prevalence of overweight and obesity in children and adults during 1980-2013: a systematic analysis for the global burden of disease study 2013. Lancet.

[15] World Health Organization (2024). Obesity and overweight. https://www.who.int/news-room/fact-sheets/detail/obesity-and-overweight.

[16] Stephenson J., Smith C.M., Kearns B., Haywood A., Bissell P. (2021). The association between obesity and quality of life: a retrospective analysis of a large-scale population-based cohort study. BMC Public Health.

[17] Afshin A., Forouzanfar M.H., Reitsma M. (2017). Health effects of overweight and obesity in 195 countries over 25 years. N Engl J Med.

[18] Maitre L., Bustamante M., Hernández-Ferrer C. (2022). Multi-omics signatures of the human early life exposome. Nat Commun.

[19] Troisi J., Belmonte F., Bisogno A. (2019). Metabolomic salivary signature of pediatric obesity related liver disease and metabolic syndrome. Nutrients.

[20] Fidalgo T.K.S., Freitas-Fernandes L.B., Angeli R. (2013). Salivary metabolite signatures of children with and without dental caries lesions. Metabolomics.

[21] Ercolini D., Francavilla R., Vannini L. (2015). From an imbalance to a new imbalance: Italian-style gluten-free diet alters the salivary microbiota and metabolome of African celiac children. Sci Rep.

[22] Cochran D., Noureldein M., Bezdeková D., Schram A., Howard R., Powers R. (2024). A reproducibility crisis for clinical metabolomics studies. TrAC Trends Anal Chem.

[23] Roth H.E., Powers R. (2022). Meta-analysis reveals both the promises and the challenges of clinical metabolomics. Cancers (Basel).

[24] Dinges S.S., Hohm A., Vandergrift L.A. (2019). Cancer metabolomic markers in urine: evidence, techniques and recommendations. Nat Rev Urol.

[25] Bar N., Korem T., Weissbrod O. (2020). A reference map of potential determinants for the human serum metabolome. Nature.

[26] Pappa E., Kousvelari E., Vastardis H. (2019). Saliva in the “Omics” era: a promising tool in paediatrics. Oral Dis.

[27] Wijnant K., Van Meulebroek L., Pomian B. (2020). Validated ultra-high-performance liquid-chromatography hybrid high-resolution mass spectrometry and laser-assisted rapid evaporative ionization mass spectrometry for salivary metabolomics. Anal Chem.

[28] Gardner A., Parkes H.G., So P.W., Carpenter G.H. (2019). Determining bacterial and host contributions to the human salivary metabolome. J Oral Microbiol.

[29] Wishart D.S. (2008). Metabolomics: applications to food science and nutrition research. Trends Food Sci Technol.

[30] Cole T.J., Lobstein T. (2012). Extended international (IOTF) body mass index cut-offs for thinness, overweight and obesity. Pediatr Obes.

[31] Laboratory of Integrative Metabolomics The Flemish adolescent MEtabolome (FAME)-study 2024. https://www.ugent.be/di/vpi/en/research/limet/famestudie.htm.

[32] Augusta De Oliveira Figueiredo R., Simola-Strö S., Rounge T.B. (2019). Cohort profile: the Finnish health in teens (Fin-HIT) study: a population-based study. Int J Epidemiol.

[33] Sallinen T., Viitasalo A., Lintu N. (2022). The effects of an 8-year individualised lifestyle intervention on food consumption and nutrient intake from childhood to adolescence: the PANIC study. J Nutr Sci.

[34] Eloranta A.-M., Sallinen T., Viitasalo A. (2021). The effects of a 2-year physical activity and dietary intervention on plasma lipid concentrations in children: the PANIC study. Eur J Nutr.

[35] Klosowska J.C., Verbeken S., Braet C. (2020). The moderating role of emotion regulation in the association between stressors with psychological and biological measures in adolescence. Psychosom Med.

[36] Roelants M., Hauspie R., Hoppenbrouwers K. (2009). References for growth and pubertal development from birth to 21 years in Flanders, Belgium. Ann Hum Biol.

[37] Nordin M., Nordin S. (2013). Psychometric evaluation and normative data of the Swedish version of the 10-item perceived stress scale. Scand J Psychol.

[38] Cohen S., Kamarck T., Mermelstein R. (1983). A global measure of perceived stress. J Health Soc Behav.

[39] Kovacs M. (2015). Children's depression inventory (CDI and CDI 2). Encycl Clin Psychol.

[40] Sinclair S.J., Blais M.A., Gansler D.A., Sandberg E., Bistis K., LoCicero A. (2010). Psychometric properties of the rosenberg self-esteem scale: overall and across demographic groups living within the United States. Eval Health Prof.

[41] Van Strien T., Frijters J.E.R., Bergers G.P.A., Defares P.B. (1986). The Dutch eating behavior questionnaire (DEBQ) for assessment of restrained, emotional, and external eating behavior. Int J Eat Disord.

[42] Braet C., Van Strien T. (1997). Assessment of emotional, externally induced and restrained eating behaviour in nine to twelve-year-old obese and non-obese children. Behav Res Ther.

[43] Lanfer A., Hebestreit A., Ahrens W. (2011). Reproducibility of food consumption frequencies derived from the Children's eating habits questionnaire used in the IDEFICS study. Int J Obes.

[44] Bel-Serrat S., Mouratidou T., Pala V. (2014). Relative validity of the Children's Eating Habits Questionnaire-food frequency section among young European children: the IDEFICS study. Public Health Nutr.

[45] Collins E.M. (1991). Promoting healthy body image through the comprehensive school health program. J Health Educ.

[46] Leppänen M.H., Lehtimäki A.V., Roos E., Viljakainen H. (2022). Body mass index, physical activity, and body image in adolescents. Children.

[47] Currie C., Gabhainn N.S., Godeau E. (2008).

[48] Saari A., Sankilampi U., Hannila M.L., Kiviniemi V., Kesseli K., Dunkel L. (2011). New Finnish growth references for children and adolescents aged 0 to 20 years: length/height-for-age, weight-for-length/height, and body mass index-for-age. Ann Med.

[49] Partinen M., Gislason T. (1995). Basic nordic sleep questionnaire (BNSQ): a quantitated measure of subjective sleep complaints. J Sleep Res.

[50] Väistö J., Eloranta A.M., Viitasalo A. (2014). Physical activity and sedentary behaviour in relation to cardiometabolic risk in children: cross-sectional findings from the physical activity and nutrition in children (PANIC) study. Int J Behav Nutr Phys Act.

[51] Rastas M., Seppaenen R., Knuts L.-R., Karvetti R.-L., Varo P. (1989).

[52] Laurent J., Catanzaro S.J., Rudolph K.D. (1999). A measure of positive and negative affect for children: scale development and preliminary validation. Psychol Assess.

[53] Schymanski E.L., Jeon J., Gulde R. (2014). Identifying small molecules via high resolution mass spectrometry: communicating confidence. Environ Sci Technol.

[54] Vangeenderhuysen P., Vynck M., Pomian B. (2025). Automated integration and quality assessment of chromatographic peaks in LC-MS-Based metabolomics and lipidomics using TARDIS. Anal Chem.

[55] Broadhurst D., Goodacre R., Reinke S.N. (2018). Guidelines and considerations for the use of system suitability and quality control samples in mass spectrometry assays applied in untargeted clinical metabolomic studies. Metabolomics.

[56] Jose S., Thermo Fisher Scientific Technical Publications C (2023).

[57] Louail P., Brunius C., Garcia-Aloy M. (2025). Xcms at 20 and still in peak form: anchoring a complete metabolomics data preprocessing and analysis software ecosystem. Bolzano Italy.

[58] Dunn W.B., Broadhurst D., Begley P. (2011). Procedures for large-scale metabolic profiling of serum and plasma using gas chromatography and liquid chromatography coupled to mass spectrometry. Nat Protoc.

[59] Wang Q., Garrity G.M., Tiedje J.M., Cole J.R. (2007). Naïve Bayesian classifier for rapid assignment of rRNA sequences into the new bacterial taxonomy. Appl Environ Microbiol.

[60] Räisänen L., Agrawal N., Mathew B., Kääriäinen S., Kolho K.L., Viljakainen H. (2023). Pre-diagnostic saliva microbiota of school-aged children who developed type 1 diabetes or inflammatory bowel diseases. Int J Mol Sci.

[61] Raju S.C., Lagström S., Ellonen P. (2019). Gender-specific associations between saliva microbiota and body size. Front Microbiol.

[62] Schloss P.D., Westcott S.L., Ryabin T. (2009). Introducing mothur: open-source, platform-independent, community-supported software for describing and comparing microbial communities. Appl Environ Microbiol.

[63] Quast C., Pruesse E., Yilmaz P. (2013). The SILVA ribosomal RNA gene database project: improved data processing and web-based tools. Nucleic Acids Res.

[64] Oren A., Arahal D.R., Göker M., Moore E.R.B., Rossello-Mora R., Sutcliffe I.C. (2023). International code of nomenclature of prokaryotes. Prokaryotic code (2022 revision). Int J Syst Evol Microbiol.

[65] Parte A.C., Carbasse J.S., Meier-Kolthoff J.P., Reimer L.C., Göker M. (2020). List of prokaryotic names with standing in nomenclature (LPSN) moves to the DSMZ. Int J Syst Evol Microbiol.

[66] Code of nomenclature. https://the-icsp.org/index.php/code-of-nomenclatur.

[67] Asnicar F., Berry S.E., Valdes A.M. (2021). Microbiome connections with host metabolism and habitual diet from 1,098 deeply phenotyped individuals. Nat Med.

[68] Lejeune S., Kaushik A., Parsons E.S. (2024). Untargeted metabolomic profiling in children identifies novel pathways in asthma and atopy. J Allergy Clin Immunol.

[69] Schirmer M., Stražar M., Avila-Pacheco J. (2024). Linking microbial genes to plasma and stool metabolites uncovers host-microbial interactions underlying ulcerative colitis disease course. Cell Host Microbe.

[70] Alexander J.L., Mullish B.H., Danckert N.P. (2023). The gut microbiota and metabolome are associated with diminished COVID-19 vaccine-induced antibody responses in immunosuppressed inflammatory bowel disease patients. EBioMedicine.

[71] Moore S.C., Playdon M.C., Sampson J.N. (2018). A metabolomics analysis of body mass index and postmenopausal breast cancer risk. J Natl Cancer Inst.

[72] Pang Z., Lu Y., Zhou G. (2024). MetaboAnalyst 6.0: towards a unified platform for metabolomics data processing, analysis and interpretation. Nucleic Acids Res.

[73] Ballenberger N., Lluis A., von Mutius E., Illi S., Schaub B. (2012). Novel statistical approaches for non-normal censored immunological data: analysis of cytokine and gene expression data. PLoS One.

[74] Vangeenderhuysen P., Van Arnhem J., Pomian B. (2023). A dual UHPLC-HRMS-based fecal metabolomics and lipidomics analysis and automated data processing pipeline for comprehensive gut phenotyping. Anal Chem.

[75] Wallner-Liebmann S., Tenori L., Mazzoleni A. (2016). Individual human metabolic phenotype analyzed by 1H NMR of saliva samples. J Proteome Res.

[76] Walsh M.C., Brennan L., Malthouse J.P.G., Roche H.M., Gibney M.J. (2006). Effect of acute dietary standardization on the urinary, plasma, and salivary metabolomic profiles of healthy humans. Am J Clin Nutr.

[77] Li Z., Sarnat J.A., Liu K.H. (2022). Evaluation of the use of saliva metabolome as a surrogate of blood metabolome in assessing internal exposures to traffic-related air pollution. Environ Sci Technol.

[78] Bachlechner U., Floegel A., Steffen A. (2016). Associations of anthropometric markers with serum metabolites using a targeted metabolomics approach: results of the EPIC-Potsdam study. Nutr Diabetes.

[79] Carayol M., Leitzmann M.F., Ferrari P. (2017). Blood metabolic signatures of body mass index: a targeted metabolomics study in the EPIC cohort. J Proteome Res.

[80] Sugimoto M., Saruta J., Matsuki C. (2013). Physiological and environmental parameters associated with mass spectrometry-based salivary metabolomic profiles. Metabolomics.

[81] Bosman P., Pichon V., Acevedo A.C., Chardin H., Combes A. (2022). Development of analytical methods to study the salivary metabolome: impact of the sampling. Anal Bioanal Chem.

[82] Nam M., Jo S.R., Park J.H., Kim M.S. (2023). Evaluation of critical factors in the preparation of saliva sample from healthy subjects for metabolomics. J Pharm Biomed Anal.

[83] Xu Z., Yeoh Y.K., Tun H.M. (2024). Variation in the metagenomic analysis of fecal microbiome composition calls for a standardized operating approach. Microbiol Spectr.

[84] Taghizadeh N., Mohammadi S., Yousefi Z. (2023). Assessment of global histone acetylation in pediatric and adolescent obesity: correlations with SIRT1 expression and metabolic-inflammatory profiles. PLoS One.

[85] Cooke M., Leeves N., White C. (2003). Time profile of putrescine, cadaverine, indole and skatole in human saliva. Arch Oral Biol.

[86] Chu S., Chan A.K.Y., Chu C.H. (2024). Polyamines in dysbiotic oral conditions of older adults: a scoping review. Int J Mol Sci.

[87] de Lemos G.M., Resende C.M.M., Campello C.P. (2024). Is oral microbiota associated with overweight and obesity in children and adolescents? A systematic review. Crit Rev Food Sci Nutr.

[88] Radenkovic S. (2023). Investigating the effects of artificial sweeteners. Nature Rev Endocrinol.

[89] Haalck I., Székely A., Ramne S. (2024). Are we using more sugar substitutes? Wastewater analysis reveals differences and rising trends in artificial sweetener usage in Swedish urban catchments. Environ Int.

[90] Sylvetsky A.C., Welsh J.A., Brown R.J., Vos M.B. (2012). Low-calorie sweetener consumption is increasing in the United States. Am J Clin Nutr.

[91] Sylvetsky A.C., Rother K.I. (2016). Trends in the consumption of low-calorie sweeteners. Physiol Behav.

[92] Russell C., Baker P., Grimes C., Lindberg R., Lawrence M.A. (2023). Global trends in added sugars and non-nutritive sweetener use in the packaged food supply: drivers and implications for public health. Public Health Nutr.

[93] Drinking soft-drinks - european health information gateway n.d. https://gateway.euro.who.int/en/indicators/hbsc_5-drinking-soft-drinks/?utm_source=chatgpt.com#id=26218.

[94] Walton J., Wittekind A. (2023). Soft drink intake in Europe—A review of data from nationally representative food consumption surveys. Nutrients.

[95] Bian X., Chi L., Gao B., Tu P., Ru H., Lu K. (2017). The artificial sweetener acesulfame potassium affects the gut microbiome and body weight gain in CD-1 mice. PLoS One.

[96] Shou N., Rensing C., Lin Q. (2024). Acesulfame potassium induces hepatic inflammation and fatty acids accumulation via disturbance of carnitine metabolism and gut microbiota. Food Biosci.

[97] Olivier-Van Stichelen S., Rother K.I., Hanover J.A. (2019). Maternal exposure to non-nutritive sweeteners impacts progeny's metabolism and microbiome. Front Microbiol.

[98] Dao M.C., Everard A., Aron-Wisnewsky J. (2016). Akkermansia muciniphila and improved metabolic health during a dietary intervention in obesity: relationship with gut microbiome richness and ecology. Gut.

[99] Ley R.E., Turnbaugh P.J., Klein S., Gordon J.I. (2006). Human gut microbes associated with obesity. Nature.

[100] Ibi D., Suzuki F., Hiramatsu M. (2018). Effect of AceK (acesulfame potassium) on brain function under dietary restriction in mice. Physiol Behav.

[101] Lobach A.R., Roberts A., Rowland I.R. (2019). Assessing the in vivo data on low/no-calorie sweeteners and the gut microbiota. Food Chem Toxicol.

