# Supplementary material for: A repository of the salivary metabolome and its key drivers in 1436 European children
Source: eBioMedicine. 2025 Nov 11;122:106019. doi: 10.1016/j.ebiom.2025.106019 (PMC12657424; doi:10.1016/j.ebiom.2025.106019)
Supplement: Multimedia component 2 [file mmc2.docx]

**Supporting Information for**

A Repository of the Salivary Metabolome and its Key Drivers in 1,436 European Children

Ellen De Paepe^a,1^, Emile Callemeyn^a,b,^^[[1]](#footnote-2)^, Kimberly De Windt^a,c^, Kathleen Wijnant^a,c^, Vera Plekhova^a^, Beata Pomian^a^, Pablo Vangeenderhuysen^a^, Stefaan De Henauw^c^, Nathalie Michels^c^, Heli Viljakainen^d,e^, Marja H. Leppänen^d,f^, Timo A. Lakka^f,g,h^, Karolien Van De Maele^i^, Nele Baeck^j^, Ruth De Bruyne^b,k^, Sander Lefere^b,l^, Anja Geerts^b,l^, Matthijs Vynck^a^ and Lynn Vanhaecke^a,m,*^

^a^ Ghent University, Faculty of Veterinary Medicine, Department of Translational Physiology, Infectiology and Public Health, Laboratory of Integrative Metabolomics (LIMET), Belgium

^b^ Ghent University, Faculty of Medicine and Health Sciences, Department of Internal Medicine and Paediatrics, Hepatology Research Unit, Belgium

^c^ Ghent University, Faculty of Medicine and Health Sciences, Department of Public Health and Primary Care, Belgium

^d^ University of Helsinki, Faculty of Medicine, Finland

^e^ Folkhälsan Research Centre, Program for Public Health, Finland

^f^ University of Eastern Finland, School of Medicine, Institute of Biomedicine, Finland

^g^ Foundation for Research in Health Exercise and Nutrition, Kuopio Research Institute of Exercise Medicine, Finland

^h^ Department of Clinical Physiology and Nuclear Medicine, Kuopio University Hospital, Kuopio, Finland

^i^ Antwerp University Hospital, Department of Paediatrics, Belgium

^j^ General Hospital Jan Palfijn Ghent, Department of Paediatrics, Paediatric Gastroenterology, Belgium

^k^ Ghent University Hospital, Department of Paediatric Gastroenterology, Hepatology and Nutrition, Liver Research Centre Ghent, Belgium

^l^ Ghent University Hospital, Department of Gastroenterology & Hepatology, Liver Research Centre Ghent, Belgium

^m^ Queens' University Belfast, School of Biological Sciences, United Kingdom

*Corresponding author: Lynn Vanhaecke, Salisburylaan 133, 9820 Merelbeke, +32 9 264 74 51

**Email:** [Lynn.Vanhaecke@ugent.be](mailto:Lynn.Vanhaecke@ugent.be)

**This file includes:**

Tables S2, S5 to S20, S24, S25, S29 to S34, S36, S38, S39a, S40, S41a, S42 to S49,

Legends for Tables S1, S3, S4, S21 to S23, S26 to S28, S35, S37, S39b, S41b, S50, S51

**Other supporting materials for this manuscript include the following:**

Tables S1, S3, S4, S21 to S23, S26 to S28, S35, S37, S39b, S41b, S50, S51

Table S1 (separate file). Food frequency questionnaire employed in the FAME and OPERA cohorts.

Participants reported the frequency of consumption for each food item using the following response options, which were assigned numeric values from 0-3: “(almost) never” = 0, “a few times a week” = 1, “(almost) daily” = 2, and “multiple times a day” = 3. These numeric values were used for the statistical analysis (see Excel file).

Table S2. Food frequency questionnaire employed in the Fin-HIT cohort.

| **Food item** | **Extra info/Examples** |
| --- | --- |
| Chocolate and sweets |  |
| Dark bread |  |
| Sweet pastry | Bun, doughnut, Danish pastry |
| Biscuits and cookies |  |
| Pizza |  |
| Hamburger or hotdog |  |
| Milk or soured milk |  |
| Ice cream |  |
| Cooked vegetables |  |
| Fresh or grated vegetables and salads |  |
| Fruit or berries |  |
| Juice |  |
| Salty snacks |  |

Participants reported the frequency of consumption for each food item using the following response options, which were assigned numeric values from 1 to 7: “not at all” = 1, “less than once a week” = 2,“once a week” = 3, “2–4 times a week” = 4, “5–6 times a week” = 5, “once a day” = 6, and “several times a day” = 7. These numeric values were used for the statistical analysis.

Table S3 (separate file). Food record questionnaire employed in the PANIC cohort.

Dietary intake was quantified in grams per day using the Micro Nutrica® software, based on detailed four-day (four consecutive days, consisting of two week and two weekend days) food records completed by parents (see Excel file).

Table S4 (separate file). Targeted metabolites in the FAME, Fin-HIT, PANIC and OPERA cohorts.

List of the targeted metabolites used for Tier 1 identification in the UHPLC-HRMS analysis, with their detection in the exploratory cohorts FAME and Fin-HIT, and the validation cohorts OPERA and PANIC (see Excel file).

Table S5. Comparative summary of the 16S rRNA sequencing, processing and taxonomy annotation pipelines used in each cohort.

|  | **Fin-HIT** | **PANIC** |
| --- | --- | --- |
| **Year** | 2015 | 2022 |
| **Organization** | Technology Centre, Sequencing Unit, Institute for Molecular Medicine Finland (FIMM) | Technology Centre, Sequencing Unit, Institute for Molecular Medicine Finland (FIMM) |
| **Lysis step** | Bacterial cell lysis (lysozyme) + mechanical disruption (bead-beating) | Bacterial cell lysis (lysozyme) + mechanical disruption (bead-beating) |
| **Sequenced region** | V3-V4 | V3-V4 |
| **Amplification protocol** | TruSeq-tailed 1-step | TruSeq-switched tail |
| **Sequencing platform** | Illumina HiSeq1500 | Illumina MiSeq PE300 |
| **Read length** | 2×270 bp | 2×301 bp |
| **Processing pipeline** | MiSeq SOP mothur (version 1.35.1) | CLC Genomics Workbench (version 22) |
| **Reference database** | SILVA (version 119) | SILVA (version 138.1) |
| **Clustering method** | OTUs, ≥98% identity | OTUs, ≥97% identity |
| **Database classifier** | Naive Bayesian classifier | Naive Bayesian classifier |
| **Notes** | Standard classifier [1], typically implemented via mothur [2] or QIIME [3]. | Later SILVA version with improved taxonomy resolution [1,3]. |

[1] SILVA taxonomy n.d. https://www.arb-silva.de/documentation/silva-taxonomy/ (accessed August 25, 2025).

[2] Schloss PD, Westcott SL, Ryabin T, Hall JR, Hartmann M, Hollister EB, et al. Introducing mothur: open-source, platform-independent, community-supported software for describing and comparing microbial communities. Appl Environ Microbiol 2009;75:7537–41. https://doi.org/10.1128/AEM.01541-09.

[3] QIIME 2 Library n.d. https://library.qiime2.org/data-resources (accessed August 25, 2025).

[4] Wang Q, Cole JR. Updated RDP taxonomy and RDP Classifier for more accurate taxonomic classification. Microbiol Resour Announc 2024;13. https://doi.org/10.1128/mra.01063-23.

Table S6. Updated taxonomic classifications across cohorts, based on validly published names.

Taxonomic names were updated to validly published counterparts based on the List of Prokaryotic Names with Standing in Nomenclature (LPSN, accessed on 28 August 2025), in accordance with ICSP and ICNP guidelines, to improve consistency across cohorts.

| **Taxonomic level** | **Original classification** | **Updated classification** |
| --- | --- | --- |
| Phylum level | Proteobacteria | Pseudomonadota |
|  | Bacteroidetes | Bacteroidota |
|  | Firmicutes | Bacillota |
|  | Actinobacteria | Actinomycetota |
|  | Fusobacteria | Fusobacteriota |
|  | Spirochaetes | Spirochaetota |
|  | Synergistetes | Synergistota |
| Class level | Proteobacteria | Actinomycetes |
|  | Fusobacteriota | Fusobacteriia |
|  | Spirochaetes | Spirochaetia |
|  | Campylobacteria | Epsilonproteobacteria |
| Order level | Actinobacteridae | Actinomycetales |
|  | Coriobacteridae | Coriobacteriales |
|  | Corynebacteriales | Mycobacteriales |
| Family level | Coriobacteriales | Coriobacteriaceae |
|  | Actinomycetales | Actinomycetaceae |
| Other reclassifications | Epsilonproteobacteria nested in phylum Proteobacteria (now  Pseudomonadota) | Campylobacterota (phylum) |
| Order Flavobacteriales | Class Bacteroidia | Class Flavobacteriia |
| Genus *Neisseria* | Order Burkholderiales of the class Gammaproteobacteria | Order Neisseriales of the class Betaproteobacteria |
| Genus *Rothia* | Order Actinomycetales, family Actinomycetaceae | Order Micrococcales family Micrococcaceae |
| Genus *Selenomonas* | Class Clostridia, order Clostridiales | Class Negativicutes order Selenomonadales, and family Selenomonadaceae |
| Genus *Pasteurellaceae* | Order Enterobacterales | Order Pasteurellales |
| Genus *Veillonellaceae* | Class Clostridia*,* order Clostridiales | Class Negativicutes, order Veillonellales |

Table S7. Functional analysis of age-correlated salivary metabolites in the FAME cohort (*p*(γ)≤0.10).

|  | **Pathway total** | **Hits total** | **Hits significant** | **p(γ)** |
| --- | --- | --- | --- | --- |
| Butanoate metabolism | 34 | 21 | 12 | 7.70E-2 |
| Arginine and proline metabolism | 45 | 31 | 12 | 7.79E-2 |
| Aspartate and asparagine metabolism | 114 | 62 | 19 | 7.83E-2 |
| Glycine, serine, alanine and threonine metabolism | 88 | 47 | 15 | 7.90E-2 |
| Lysine metabolism | 52 | 20 | 8 | 8.01E-2 |
| Urea cycle/amino group metabolism | 85 | 41 | 13 | 8.02E-2 |
| Glutamate metabolism | 15 | 9 | 5 | 8.05E-2 |
| Methionine and cysteine metabolism | 94 | 27 | 8 | 8.99E-2 |
| Nitrogen metabolism | 6 | 5 | 3 | 9.09E-2 |
| β-alanine metabolism | 20 | 14 | 5 | 9.22E-2 |
| Pyrimidine metabolism | 70 | 33 | 9 | 9.24E-2 |
| Valine, leucine and isoleucine degradation | 65 | 19 | 6 | 9.32E-2 |
| Purine metabolism | 80 | 39 | 10 | 9.46E-2 |
| Tryptophan metabolism | 94 | 50 | 12 | 9.66E-2 |

Table S8. Functional analysis of age-correlated salivary metabolites in the Fin-HIT cohort (*p*(γ)≤0.10).

|  | **Pathway total** | **Hits total** | **Hits significant** | **p(γ)** |
| --- | --- | --- | --- | --- |
| Tryptophan metabolism | 94 | 47 | 24 | 1.01E-2 |
| Fatty acid activation | 74 | 16 | 8 | 1.06E-2 |
| Carnitine shuttle | 72 | 13 | 7 | 1.07E-2 |
| Fatty acid metabolism | 63 | 11 | 6 | 1.11E-2 |
| De novo fatty acid biosynthesis | 106 | 15 | 7 | 1.14E-2 |
| Lysine metabolism | 52 | 16 | 7 | 1.19E-2 |
| Saturated fatty acids β-oxidation | 36 | 3 | 3 | 1.24E-2 |
| ω-3 Fatty acid metabolism | 39 | 4 | 3 | 1.45E-2 |
| Glycosphingolipid metabolism | 67 | 17 | 6 | 1.65E-2 |
| Leukotriene metabolism | 92 | 26 | 8 | 1.69E-2 |
| Alkaloid biosynthesis II | 10 | 5 | 3 | 1.72E-2 |
| Vitamin B6 (pyridoxine) metabolism | 11 | 5 | 3 | 1.72E-2 |
| Glycerophospholipid metabolism | 156 | 23 | 7 | 1.90E-2 |
| Di-unsaturated fatty acid β-oxidation | 26 | 2 | 2 | 2.11E-2 |
| Glycolysis and gluconeogenesis | 49 | 11 | 4 | 2.24E-2 |
| Histidine metabolism | 33 | 16 | 5 | 2.35E-2 |
| Drug metabolism - cytochrome P450 | 53 | 21 | 6 | 2.42E-2 |
| Bile acid biosynthesis | 82 | 13 | 4 | 2.93E-2 |
| Linoleate metabolism | 46 | 25 | 6 | 3.63E-2 |
| Vitamin H (biotin) metabolism | 5 | 4 | 2 | 3.65E-2 |
| TCA cycle | 31 | 4 | 2 | 3.65E-2 |
| ω-6 Fatty acid metabolism | 55 | 4 | 2 | 3.65E-2 |
| Glycine, serine, alanine and threonine metabolism | 88 | 31 | 7 | 3.92E-2 |
| Butanoate metabolism | 34 | 17 | 4 | 4.88E-2 |
| Tyrosine metabolism | 160 | 56 | 11 | 5.33E-2 |
| Selenoamino acid metabolism | 35 | 6 | 2 | 5.53E-2 |
| Phosphatidylinositol phosphate metabolism | 59 | 7 | 2 | 6.57E-2 |
| Methionine and cysteine metabolism | 94 | 14 | 3 | 6.92E-2 |
| Squalene and cholesterol biosynthesis | 55 | 16 | 3 | 8.64E-2 |
| Alanine and aspartate metabolism | 30 | 10 | 2 | 9.94E-2 |

Table S9. Functional analysis of WtoH percentage-correlated salivary metabolites in the FAME cohort (*p*(γ)≤0.10).

|  | **Pathway total** | **Hits total** | **Hits significant** | **p(γ)** |
| --- | --- | --- | --- | --- |
| Purine metabolism | 80 | 37 | 18 | 7.41E-2 |
| Aspartate and asparagine metabolism | 114 | 60 | 21 | 7.59E-2 |
| Nitrogen metabolism | 6 | 5 | 4 | 7.84E-2 |
| Butanoate metabolism | 34 | 21 | 9 | 7.85E-2 |
| Vitamin B3 (nicotinate and nicotinamide) metabolism | 28 | 12 | 6 | 8.03E-2 |
| Tryptophan metabolism | 94 | 47 | 15 | 8.32E-2 |
| Glycolysis and gluconeogenesis | 49 | 26 | 9 | 8.73E-2 |
| Glycine, serine, alanine and threonine metabolism | 88 | 46 | 14 | 8.78E-2 |
| Pyruvate metabolism | 20 | 15 | 6 | 8.87E-2 |
| Lysine metabolism | 52 | 19 | 7 | 8.93E-2 |
| Urea cycle/amino group metabolism | 85 | 39 | 12 | 8.99E-2 |

Table S10. Functional analysis of WtoH percentage-correlated salivary metabolites in the Fin-HIT cohort (*p*(γ)≤0.10).

|  | **Pathway total** | **Hits total** | **Hits significant** | **p(γ)** |
| --- | --- | --- | --- | --- |
| Lysine metabolism | 52 | 16 | 6 | 1.55E-2 |
| De novo fatty acid biosynthesis | 106 | 15 | 5 | 2.15E-2 |
| Butanoate metabolism | 34 | 17 | 5 | 2.71E-2 |
| Glycosphingolipid metabolism | 67 | 17 | 5 | 2.71E-2 |
| Saturated fatty acids β-oxidation | 36 | 3 | 2 | 2.88E-2 |
| Linoleate metabolism | 46 | 25 | 6 | 3.77E-2 |
| Drug metabolism - cytochrome P450 | 53 | 21 | 5 | 4.28E-2 |
| Vitamin E metabolism | 54 | 16 | 4 | 4.46E-2 |
| Fatty acid activation | 74 | 16 | 4 | 4.46E-2 |
| Alkaloid biosynthesis II | 10 | 5 | 2 | 4.65E-2 |
| Porphyrin metabolism | 43 | 5 | 2 | 4.65E-2 |
| Fatty acid metabolism | 63 | 11 | 3 | 4.80E-2 |
| Glycerophospholipid metabolism | 156 | 23 | 5 | 5.29E-2 |
| Aspartate and asparagine metabolism | 114 | 34 | 7 | 5.34E-2 |
| Tyrosine metabolism | 160 | 56 | 11 | 5.63E-2 |
| Putative anti-inflammatory metabolites formation from EPA | 27 | 6 | 2 | 5.65E-2 |
| Xenobiotics metabolism | 110 | 18 | 4 | 5.65E-2 |

Table S11. Functional analysis of BMIz-correlated salivary metabolites in the FAME cohort (*p*(γ)≤0.10).

|  | **Pathway total** | **Hits total** | **Hits significant** | **p(γ)** |
| --- | --- | --- | --- | --- |
| Butanoate metabolism | 34 | 21 | 13 | 7.79E-2 |
| Aspartate and asparagine metabolism | 114 | 60 | 23 | 7.84E-2 |
| Purine metabolism | 80 | 37 | 16 | 7.85E-2 |
| Arginine and proline metabolism | 45 | 31 | 13 | 7.97E-2 |
| Propanoate metabolism | 31 | 15 | 8 | 7.98E-2 |
| Vitamin B3 (nicotinate and nicotinamide) metabolism | 28 | 12 | 7 | 7.98E-2 |
| Valine, leucine and isoleucine degradation | 65 | 19 | 9 | 8.04E-2 |
| Tryptophan metabolism | 94 | 47 | 17 | 8.06E-2 |
| Glycine, serine, alanine and threonine metabolism | 88 | 46 | 16 | 8.23E-2 |
| Glutamate metabolism | 15 | 9 | 5 | 8.51E-2 |
| Urea cycle/amino group metabolism | 85 | 39 | 13 | 8.70E-2 |
| Lysine metabolism | 52 | 19 | 7 | 9.36E-2 |
| Nitrogen metabolism | 6 | 5 | 3 | 9.89E-2 |

Table S12. Functional analysis of BMIz-correlated salivary metabolites in the Fin-HIT cohort (*p*(γ)≤0.10).

|  | **Pathway total** | **Hits total** | **Hits significant** | **p(γ)** |
| --- | --- | --- | --- | --- |
| Lysine metabolism | 52 | 16 | 7 | 1.32E-2 |
| Glycerophospholipid metabolism | 156 | 23 | 7 | 2.08E-2 |
| Aspartate and asparagine metabolism | 114 | 34 | 9 | 2.40E-2 |
| Drug metabolism - cytochrome P450 | 53 | 21 | 6 | 2.63E-2 |
| Butanoate metabolism | 34 | 17 | 5 | 2.85E-2 |
| Saturated fatty acids β-oxidation | 36 | 3 | 2 | 3.04E-2 |
| Tryptophan metabolism | 94 | 47 | 11 | 3.06E-2 |
| Carnitine shuttle | 72 | 13 | 4 | 3.16E-2 |
| Vitamin H (biotin) metabolism | 5 | 4 | 2 | 3.88E-2 |
| ω-3 Fatty acid metabolism | 39 | 4 | 2 | 3.88E-2 |
| De novo fatty acid biosynthesis | 106 | 15 | 4 | 4.08E-2 |
| Fatty acid activation | 74 | 16 | 4 | 4.60E-2 |
| Alkaloid biosynthesis II | 10 | 5 | 2 | 4.81E-2 |
| Porphyrin metabolism | 43 | 5 | 2 | 4.81E-2 |
| Pyruvate metabolism | 20 | 11 | 3 | 4.94E-2 |
| Arginine and proline metabolism | 45 | 19 | 4 | 6.43E-2 |
| Vitamin E metabolism | 54 | 16 | 3 | 8.98E-2 |
| Propanoate metabolism | 31 | 9 | 2 | 9.10E-2 |

Table S13. Functional analysis of fat percentage-correlated salivary metabolites in the FAME cohort (*p*(γ)≤0.10).

|  | **Pathway total** | **Hits total** | **Hits significant** | **p(γ)** |
| --- | --- | --- | --- | --- |
| Tryptophan metabolism | 94 | 47 | 20 | 7.50E-2 |
| Aspartate and asparagine metabolism | 114 | 60 | 18 | 8.01E-2 |
| Butanoate metabolism | 34 | 21 | 7 | 9.07E-2 |
| Biopterin metabolism | 22 | 9 | 4 | 9.26E-2 |
| Valine, leucine and isoleucine degradation | 65 | 19 | 6 | 9.86E-2 |
| Lysine metabolism | 52 | 19 | 6 | 9.86E-2 |

Table S14. Functional analysis of CDI-2-correlated salivary metabolites in the FAME cohort (*p*(γ)≤0.10).

|  | **Pathway total** | **Hits total** | **Hits significant** | **p(γ)** |
| --- | --- | --- | --- | --- |
| Butanoate metabolism | 34 | 21 | 7 | 7.69E-2 |
| Aspartate and asparagine metabolism | 114 | 60 | 14 | 7.70E-2 |
| Urea cycle/amino group metabolism | 85 | 39 | 10 | 7.80E-2 |
| β-Alanine metabolism | 20 | 13 | 5 | 7.88E-2 |
| Arginine and proline metabolism | 45 | 31 | 8 | 8.08E-2 |
| Glycine, serine, alanine and threonine metabolism | 88 | 46 | 10 | 8.41E-2 |
| Lysine metabolism | 52 | 19 | 5 | 9.03E-2 |

Table S15. Functional analysis of PSS salivary metabolites in the FAME cohort (*p*(γ)≤0.10).

|  | **Pathway total** | **Hits total** | **Hits significant** | **p(γ)** |
| --- | --- | --- | --- | --- |
| Glutamate metabolism | 15 | 9 | 5 | 7.50E-2 |
| Carbon fixation | 10 | 9 | 5 | 7.50E-2 |
| Arginine and proline metabolism | 45 | 31 | 10 | 7.54E-2 |
| Lysine metabolism | 52 | 19 | 7 | 7.66E-2 |
| Methionine and cysteine metabolism | 94 | 25 | 8 | 7.80E-2 |
| Pyrimidine metabolism | 70 | 30 | 9 | 7.82E-2 |
| Butanoate metabolism | 34 | 21 | 7 | 7.89E-2 |
| Alanine and aspartate metabolism | 30 | 21 | 7 | 7.89E-2 |
| Glycolysis and gluconeogenesis | 49 | 26 | 8 | 7.91E-2 |
| Glyoxylate and dicarboxylate metabolism | 12 | 9 | 4 | 8.24E-2 |
| Urea cycle/amino group metabolism | 85 | 39 | 10 | 8.25E-2 |
| Valine, leucine and isoleucine degradation | 65 | 19 | 6 | 8.34E-2 |
| Glutathione metabolism | 19 | 5 | 3 | 8.37E-2 |
| Ascorbate (Vitamin C) and aldarate metabolism | 29 | 20 | 6 | 8.55E-2 |
| Purine metabolism | 80 | 37 | 9 | 8.76E-2 |
| Histidine metabolism | 33 | 17 | 5 | 9.13E-2 |
| Fatty acid metabolism | 63 | 17 | 5 | 9.13E-2 |
| Vitamin B3 (nicotinate and nicotinamide) metabolism | 28 | 12 | 4 | 9.24E-2 |
| TCA cycle | 31 | 12 | 4 | 9.24E-2 |
| β-Alanine metabolism | 20 | 13 | 4 | 9.64E-2 |

Table S16. Functional analysis of RSES-correlated salivary metabolites in the FAME cohort (*p*(γ)≤0.10).

|  | **Pathway total** | **Hits total** | **Hits significant** | **p(γ)** |
| --- | --- | --- | --- | --- |
| Arginine and proline metabolism | 45 | 31 | 7 | 8.40E-2 |

Table S17. Functional analysis of RSES-correlated salivary metabolites in the Fin-HIT cohort (*p*(γ)≤0.10).

|  | **Pathway total** | **Hits total** | **Hits significant** | **p(γ)** |
| --- | --- | --- | --- | --- |
| Lysine metabolism | 52 | 16 | 9 | 1.15E-2 |
| De novo fatty acid biosynthesis | 106 | 15 | 7 | 1.33E-2 |
| Fatty acid activation | 74 | 16 | 6 | 1.88E-2 |
| Glycerophospholipid metabolism | 156 | 23 | 7 | 2.48E-2 |
| CoA catabolism | 7 | 3 | 2 | 3.28E-2 |
| Glycosphingolipid metabolism | 67 | 17 | 5 | 3.38E-2 |
| N-Glycan biosynthesis | 48 | 4 | 2 | 4.24E-2 |
| Vitamin B5 - CoA biosynthesis from pantothenate | 12 | 4 | 2 | 4.24E-2 |
| 3-Oxo-10R-octadecatrienoate β-oxidation | 27 | 10 | 3 | 4.90E-2 |
| Leukotriene metabolism | 92 | 26 | 6 | 5.35E-2 |
| Fatty acid metabolism | 63 | 11 | 3 | 5.69E-2 |
| Biopterin metabolism | 22 | 6 | 2 | 6.42E-2 |
| Aspartate and asparagine metabolism | 114 | 34 | 7 | 6.97E-2 |
| Carnitine shuttle | 72 | 13 | 3 | 7.43E-2 |
| Arginine and proline metabolism | 45 | 19 | 4 | 7.67E-2 |
| Linoleate metabolism | 46 | 25 | 5 | 8.02E-2 |
| Urea cycle/amino group metabolism | 85 | 27 | 5 | 9.55E-2 |

Table S18. Functional analysis of emotional eating-correlated salivary metabolites in the FAME cohort (*p*(γ)≤0.10).

|  | **Pathway total** | **Hits total** | **Hits significant** | **p(γ)** |
| --- | --- | --- | --- | --- |
| Urea cycle/amino group metabolism | 85 | 39 | 13 | 7.14E-2 |
| Glycosphingolipid biosynthesis - ganglioseries | 62 | 8 | 5 | 7.18E-2 |
| Chondroitin sulphate degradation | 37 | 5 | 4 | 7.20E-2 |
| Heparan sulphate degradation | 34 | 5 | 4 | 7.20E-2 |
| Arginine and proline metabolism | 45 | 31 | 10 | 7.43E-2 |
| De novo fatty acid biosynthesis | 106 | 28 | 9 | 7.57E-2 |
| Alanine and aspartate metabolism | 30 | 21 | 7 | 7.85E-2 |
| Keratan sulphate degradation | 68 | 5 | 3 | 8.28E-2 |
| Nitrogen metabolism | 6 | 5 | 3 | 8.28E-2 |
| Lysine metabolism | 52 | 19 | 6 | 8.41E-2 |
| N-Glycan degradation | 16 | 6 | 3 | 8.81E-2 |
| Histidine metabolism | 33 | 17 | 5 | 9.35E-2 |
| Sialic acid metabolism | 107 | 28 | 7 | 9.53E-2 |
| Glycerophospholipid metabolism | 156 | 34 | 8 | 9.73E-2 |
| Urea cycle/amino group metabolism | 85 | 39 | 13 | 7.14E-2 |

Table S19. Functional analysis of external eating-correlated salivary metabolites in the FAME cohort (*p*(γ)≤0.10).

|  | **Pathway total** | **Hits total** | **Hits significant** | **p(γ)** |
| --- | --- | --- | --- | --- |
| Phosphatidylinositol phosphate metabolism | 59 | 14 | 3 | 9.46E-2 |

Table S20. Functional analysis of restrained eating-correlated salivary metabolites in the FAME cohort (*p*(γ)≤0.10).

|  | **Pathway total** | **Hits total** | **Hits significant** | **p(γ)** |
| --- | --- | --- | --- | --- |
| Arginine and proline metabolism | 45 | 31 | 14 | 7.47E-2 |
| Urea cycle/amino group metabolism | 85 | 39 | 15 | 7.62E-2 |
| Lysine metabolism | 52 | 19 | 9 | 7.65E-2 |
| Glycolysis and gluconeogenesis | 49 | 26 | 11 | 7.67E-2 |
| Butanoate metabolism | 34 | 21 | 9 | 7.85E-2 |
| Galactose metabolism | 41 | 26 | 10 | 8.03E-2 |
| Glyoxylate and dicarboxylate metabolism | 12 | 9 | 5 | 8.11E-2 |
| Glutamate metabolism | 15 | 9 | 5 | 8.11E-2 |
| Glycerophospholipid metabolism | 156 | 34 | 12 | 8.11E-2 |
| Glycine, serine, alanine and threonine metabolism | 88 | 46 | 15 | 8.18E-2 |
| Aspartate and asparagine metabolism | 114 | 60 | 17 | 9.09E-2 |
| Glutathione metabolism | 19 | 5 | 3 | 9.47E-2 |
| Methionine and cysteine metabolism | 94 | 25 | 8 | 9.58E-2 |
| β-Alanine metabolism | 20 | 13 | 5 | 9.63E-2 |

Table S21 (separate file). Targeted salivary metabolites and their (borderline) significant correlations (*q*≤0.20) with BMI*z*, body fat percentage (BF%), and waist-to-height (WtoH) ratio, along with (borderline) significant (*q*≤0.20) univariate differences (pairwise or multiple comparisons) between weight categories.

Pairwise comparisons are based on BMI*z* and include healthy weight (HW) *vs.* overweight (including obesity) (OW), while multiple comparisons distinguish between healthy weight (HW), overweight (OW), and obesity (OB). Results are presented for the FAME and Fin-HIT cohorts, alongside metabolite associations validated in the PANIC and OPERA cohorts. Borderline significant correlations and univariate differences (***q*≤0.05, *0.05<*q*≤0.20) are highlighted in bold (see Excel file).

Table S22a (separate file). Targeted metabolites significantly different (*q*≤0.05) between weight classes (healthy weight *vs.* overweight and obesity) in FAME, with median QC-normalised peak area values (IQR).

(see Excel file)

Table S22b (separate file). Targeted metabolites borderline significantly different (0.05<*q*≤0.20) between weight classes (healthy weight *vs.* overweight and obesity) in FAME, with median QC-normalised peak area values (IQR).

(see Excel file)

Table S23a (separate file). Targeted metabolites with an overall significant difference (*q*≤0.05) between weight classes (healthy weight *vs.* overweight *vs.* obesity) in FAME, with median QC-normalised peak area values (IQR).

Different superscript letters (a, b, c) indicate (borderline) statistically significant pairwise differences between groups (*q≤*0.20) (see Excel file).

Table S23b (separate file). Targeted metabolites with an overall borderline significant difference (*q*≤0.20) between weight classes (healthy weight *vs.* overweight *vs.* obesity) in FAME, with median QC-normalised peak area values (IQR).

Different superscript letters (a, b, c) indicate (borderline) statistically significant pairwise differences between groups (*q≤*0.20) (see Excel file).

Table S24a. Targeted metabolites significantly different (*q*≤0.05) between weight classes (healthy weight *vs.* overweight and obesity) in Fin-HIT, with median QC-normalised peak area values (IQR).

| **Metabolite Name** | **Statistic** | **Healthy weight**  **(n=234)** | **Overweight (incl. obesity)**  **(n=240)** | ***q*** |
| --- | --- | --- | --- | --- |
| N-Acetylvaline | Wilcoxon | 0.90 (0.50) | 1.07 (0.51) | 4.97E-2 |

Table S24b. Targeted metabolites borderline significantly different (0.05<*q*≤0.20) between weight classes (healthy weight *vs.* overweight and obesity) in Fin-HIT, with median QC-normalised peak area values (IQR).

| **Metabolite Name** | **Statistic** | **Healthy weight**  **(n=234)** | **Overweight (incl. obesity)**  **(n=240)** | ***q*** |
| --- | --- | --- | --- | --- |
| L-Glutamic acid | Wilcoxon | 0.72 (0.41) | 0.83 (0.44) | 9.12E-2 |
| N-Acetylleucine | Wilcoxon | 0.96 (0.77) | 1.13 (0.70) | 9.12E-2 |
| 3-Indolepropionic acid | Wilcoxon | 0.77 (1.09) | 0.97 (1.29) | 1.62E-1 |

Table S25a. Targeted metabolites with an overall significant difference (*q*≤0.05) in the multiple comparison between weight categories (healthy weight *vs.* overweight *vs.* obesity) in Fin-HIT, with median QC-normalised peak area values (IQR).

| **Metabolite Name** | **Statistic** | **Healthy weight (n=234)** | **Overweight (n=189)** | **Obesity**  **(n=51)** | ***q*** |
| --- | --- | --- | --- | --- | --- |
| N-Acetylvaline | Kruskal-Wallis | 0.90 (0.50)^b^ | 1.09 (0.47)^a^ | 0.99 (0.46)^b^ | 8.01E-3 |
| 4-Hydroxy-benzaldehyde | Kruskal-Wallis | 0.87 (0.39)^b^ | 0.80 (0.42)^c^ | 1.06 (0.62)^a^ | 8.12E-3 |
| N6-Acetyllysine | ANOVA | 0.93 (0.46)^b^ | 0.92 (0.41)^b^ | 1.10 (0.40)^a^ | 1.31E-3 |
| N-Acetylleucine | Kruskal-Wallis | 0.96 (0.77)^b^ | 1.20 (0.62)^a^ | 1.01 (0.70)^b^ | 1.33E-2 |
| L,L-Cyclo(leucylprolyl) | Kruskal-Wallis | 0.89 (0.65) | 0.81 (0.48) | 1.03 (0.65) | 1.33E-2 |
| L-Aspartyl-L-Phenylalanine | ANOVA | 0.81 (0.56)^b^ | 0.78 (0.52)^b^ | 1.01 (0.52)^a^ | 1.99E-2 |
| L-Tryptophan | Kruskal-Wallis | 1.01 (0.38)^b^ | 0.95 (0.31)^c^ | 1.14 (0.39)^a^ | 2.82E-2 |
| L-Methionine | Kruskal-Wallis | 0.86 (0.63)^a^ | 0.75 (0.55)^b^ | 1.00 (0.79)^a^ | 3.83E-2 |
| N-Acetyltryptophan | Kruskal-Wallis | 1.04 (0.54)^b^ | 1.16 (0.57)^a^ | 0.96 (0.53)^c^ | 3.83E-2 |
| L-Leucine | Kruskal-Wallis | 0.80 (0.69)^b^ | 0.70 (0.61)^b^ | 1.03 (0.80)^a^ | 3.83E-2 |
| Imidazolepropionic acid | Kruskal-Wallis | 0.44 (0.86)^b^ | 0.58 (1.01)^b^ | 0.85 (1.36)^a^ | 4.18E-2 |
| Leucylglycine | Kruskal-Wallis | 0.97 (0.54)^a^ | 0.88 (0.46)^b^ | 1.04 (0.63)^a^ | 4.76E-2 |

Different superscript letters (a, b, c) indicate (borderline) statistically significant pairwise differences between groups (*q≤*0.20).

Table S25b. Targeted metabolites with an overall borderline significant difference (0.05<*q*≤0.20) in the multiple comparison between weight categories (healthy weight *vs.* overweight *vs.* obesity) in Fin-HIT, with median QC-normalised peak area values (IQR).

| **Metabolite Name** | **Statistic** | **Healthy weight (n=234)** | **Overweight (n=189)** | **Obesity**  **(n=51)** | ***q*** |
| --- | --- | --- | --- | --- | --- |
| Putrescine | Kruskal-Wallis | 0.56 (1.24)^b^ | 0.50 (1.15)^b^ | 1.07 (2.03)^a^ | 5.40E-2 |
| L-Glutamic acid | Kruskal-Wallis | 0.72 (0.41)^b^ | 0.84 (0.51)^a^ | 0.83 (0.33)^a^ | 6.01E-2 |
| 3-Indoleacetic acid | Kruskal-Wallis | 0.71 (0.98)^b^ | 0.75 (0.90)^b^ | 0.98 (1.63)^a^ | 8.01E-2 |
| Saccharin | Kruskal-Wallis | 0.13 (0.55)^a^ | 0.12 (0.23)^b^ | 0.11 (0.07)^b^ | 8.10E-2 |

Different superscript letters (a, b, c) indicate (borderline) statistically significant pairwise differences between groups (*q≤*0.20).

Table S26a (separate file). Targeted metabolites significantly different (*q*≤0.05) between weight classes (healthy weight *vs.* overweight and obesity) in PANIC, with median QC-normalised peak area values (IQR).

(see Excel file)

Table S26b (separate file). Targeted metabolites borderline significantly different (0.05<*q*≤0.20) between weight classes (healthy weight *vs.* overweight and obesity) in PANIC, with median QC-normalised peak area values (IQR).

(see Excel file)

Table S27a (separate file). Targeted metabolites with an overall significant difference (*q*≤0.05) between weight classes (healthy weight *vs.* overweight *vs.* obesity) in PANIC, with median QC-normalised peak area values (IQR).

Different superscript letters (a, b, c) indicate (borderline) statistically significant pairwise differences between groups (*q≤*0.20) (see Excel file).

Table S27b (separate file). Targeted metabolites with an overall borderline significant difference (*q*≤0.20) between weight classes (healthy weight *vs.* overweight *vs.* obesity) in PANIC, with median QC-normalised peak area values (IQR).

Different superscript letters (a, b, c) indicate (borderline) statistically significant pairwise differences between groups (*q≤*0.20) (see Excel file).

Table S28 (separate file). Targeted salivary metabolites with (borderline) significant correlations (*q*≤0.20) with mental wellbeing parameters, along with univariate (borderline) significant differences (*q*≤0.20) (pairwise or multiple comparisons) based on mental health status.

Correlations of targeted salivary metabolites with mental wellbeing parameters - including the Child Depression Inventory-2 (CDI-2), emotional eating (EE), Perceived Stress Scale (PSS), restrained eating (RE), Rosenberg Self-Esteem Scale (RSES), external eating (ExtE), body image (BI), weekday sleep quality (SleepWk), weekend sleep quality (SleepWknd), hair cortisol levels (Cort), sleep, and Negative Affect Schedule for Children (PANAS-C) - along with univariate differences (pairwise or multiple comparisons) based on mental health status.

Stress levels were determined using a psychological stress *z-*score. For the FAME cohort, the *z-*scores of CDI-2 and PSS were summed, and RSES subtracted (as higher RSES scores indicate better mental well-being). In the Fin-HIT cohort, the absolute value of the *z-*score-transformed Collins Childhood Body Rating Scale score was used, with RSES subtracted. For the PANIC cohort, only sleep was used. For the OPERA cohort, the *z-*scores of PSS, CDI-2, and negative PANAS-C were combined.

Pairwise comparisons were conducted for low stress (lowest 50%) versus high stress (highest 50%), while multiple comparisons included low stress (lowest 25%), moderate stress (25–75%), and high stress (highest 25%). Results for the FAME and Fin-HIT cohorts are presented alongside metabolite correlations in the validation cohorts (PANIC and OPERA). Significant correlations and univariate differences (***q*≤0.05, *0.05<*q*≤0.20) are highlighted in bold (see Excel file).

Table S29. Targeted metabolites borderline significantly (*q*≤0.20) different in the pairwise comparison between stress status in FAME, with median QC-normalised peak area values (IQR).

| **Metabolite Name** | **Statistic** | **Low stress**  **(n=222)** | **High stress**  **(n=221)** | ***q*** |
| --- | --- | --- | --- | --- |
| N-Acetylmethionine | Wilcoxon | 0.15 (0.54) | 0.29 (1.16) | 1.94E-1 |

Stress levels were calculated based on a psychological stress *z-*score, which included *z-*score-transformed CDI-2 and PSS scores, which were summed, and the *z-*score-transformed RSES score, which was subtracted. Low stress included the 50% lowest stress *z-*scores, while high stress included the 50% highest *z-*scores.

Table S30. Targeted metabolites borderline significantly (*q*≤0.20) different in the multiple comparison between stress status in FAME, with median QC-normalised peak area values (IQR).

| **Metabolite Name** | **Statistic** | **Low stress**  **(n=113)** | **Moderate stress**  **(n=219)** | **High stress**  **(n=111)** | ***q*** |
| --- | --- | --- | --- | --- | --- |
| Isovaleryl-L-carnitine/  2-methylbutyryl-L-carnitine | Kruskal-Wallis | 0.95 (1.36)^a^ | 0.86 (1.30)^a^ | 0.62 (0.86)^b^ | 1.39E-1 |
| L-Arginine | Kruskal-Wallis | 0.68 (0.74)^a^ | 0.64 (0.59)^a^ | 0.50 (0.51)^b^ | 1.81E-1 |
| 1-Aminocyclopropane-1-carboxylic acid | Kruskal-Wallis | 0.94 (0.70) | 0.83 (0.53) | 1.00 (0.67) | 2.00E-1 |

Stress levels were calculated based on a psychological stress *z-*score, which included *z-*score-transformed CDI-2 and PSS scores, which were summed, and the *z-*score-transformed RSES score, which was subtracted. Low stress included the 25% lowest stress *z-*scores, while high stress included the 25% highest *z-*scores. Moderate stress levels included participants in the 25-75% *z-*score range. Different superscript letters (a, b, c) indicate statistically significant differences between groups (*q≤*0.20).

Table S31a. Targeted metabolites significantly (*q*≤0.05) different in the pairwise comparison between stress levels in OPERA, with median QC-normalised peak area values (IQR).

| **Metabolite Name** | **Statistic** | **Low stress**  **(n=66)** | **High stress**  **(n=65)** | ***q*** |
| --- | --- | --- | --- | --- |
| 4-Hydroxybenzaldehyde | Wilcoxon | 1.23 (1.12) | 0.81 (0.49) | 1.58E-3 |
| 2,6-Diaminopimelic acid | Wilcoxon | 0.39 (1.05) | 0.10 (0.42) | 4.45E-3 |
| Hypoxanthine | ANOVA | 0.95 (0.63) | 0.70 (0.52) | 6.29E-3 |
| Xanthine | Wilcoxon | 0.81 (1.10) | 0.43 (0.70) | 7.90E-3 |
| 5-Oxo-L-prolyl-L-proline | Wilcoxon | 1.01 (1.68) | 0.47 (0.85) | 1.81E-2 |
| N6-Acetyllysine | Wilcoxon | 0.60 (0.67) | 0.35 (0.42) | 2.22E-2 |
| β-Hydroxyisovaleric acid | ANOVA | 1.13 (0.66) | 1.02 (0.51) | 3.29E-2 |
| Thymine | Wilcoxon | 0.82 (0.98) | 0.54 (0.42) | 3.35E-2 |
| L-Proline | Wilcoxon | 0.99 (0.95) | 0.54 (0.82) | 3.85E-2 |
| 3-Indolepropionic acid | Wilcoxon | 1.14 (1.22) | 0.56 (0.78) | 3.85E-2 |
| 2-Ethyl-2-hydroxybutyric acid | Wilcoxon | 1.18 (0.72) | 0.95 (0.51) | 4.72E-2 |

Stress levels were calculated based on a psychological stress *z-*score, which was the sum of the *z-*score-transformed PSS, CDI-2, and the negative affect items from the PANAS-C. Low stress included the 50% lowest stress *z-*scores, while high stress included the 50% highest *z-*scores.

Table S31b. Targeted metabolites borderline significantly (0.05<*q*≤0.20) different in the pairwise comparison between stress levels in OPERA, with median QC-normalised peak area values (IQR).

| **Metabolite Name** | **Statistic** | **Low stress**  **(n=66)** | **High stress**  **(n=65)** | ***q*** |
| --- | --- | --- | --- | --- |
| N6,N6,N6-Trimethyllysine | Wilcoxon | 1.05 (0.82) | 0.83 (0.58) | 5.79E-2 |
| N,N-Dimethylarginine | ANOVA | 1.18 (0.72) | 0.82 (0.72) | 5.79E-2 |
| N-Acetylmethionine | Wilcoxon | 0.80 (1.24) | 0.42 (1.07) | 7.44E-2 |
| L-Leucine | Wilcoxon | 1.24 (1.08) | 0.83 (1.09) | 9.23E-2 |
| 3-Indoleacetic acid | Wilcoxon | 0.88 (1.85) | 0.56 (0.93) | 9.23E-2 |
| N-Formyl-methionine | Wilcoxon | 1.27 (1.06) | 0.92 (1.07) | 9.44E-2 |
| Caffeine | Wilcoxon | 0.29 (0.66) | 0.46 (1.09) | 1.06E-1 |
| L-Tryptophan | Wilcoxon | 1.15 (1.35) | 0.80 (1.01) | 1.07E-1 |
| Putrescine | ANOVA | 1.02 (0.88) | 0.90 (0.65) | 1.54E-1 |
| 2-Picolinic acid/nicotinic acid | Wilcoxon | 1.04 (0.87) | 0.89 (0.85) | 1.54E-1 |
| Histamine | Wilcoxon | 0.80 (1.53) | 0.31 (1.22) | 1.58E-1 |
| L-Arginine | ANOVA | 0.78 (0.39) | 0.71 (0.39) | 1.91E-1 |
| Acesulfame K | Wilcoxon | 0.00 (0.10) | 0.01 (0.46) | 1.91E-1 |
| 2-Hydroxyisocaproic acid/2-hydroxyhexanoic acid | Wilcoxon | 1.08 (0.93) | 0.90 (0.69) | 1.91E-1 |
| L-Methionine | Wilcoxon | 0.99 (0.99) | 0.88 (1.00) | 1.91E-1 |
| D-Pantothenic acid | Wilcoxon | 1.15 (1.30) | 0.81 (1.52) | 1.91E-1 |

Stress levels were calculated based on a psychological stress *z-*score, which was the sum of the *z-*score-transformed PSS, CDI-2, and negative affect items from the PANAS-C. Low stress included the 50% lowest stress *z-*scores, while high stress included the 50% highest *z-*scores.

Table S32a. Targeted metabolites with an overall significant (*q*≤0.05) difference in the multiple comparison between stress levels in OPERA, with median QC-normalised peak area values (IQR).

| **Metabolite Name** | **Statistic** | **Low stress**  **(n=33)** | **Moderate stress**  **(n=65)** | **High stress**  **(n=33)** | ***q*** |
| --- | --- | --- | --- | --- | --- |
| 2,6-Diaminopimelic acid | Kruskal-Wallis | 0.23 (0.86)^a^ | 0.34 (0.79)^a^ | 0.05 (0.24)^b^ | 3.64E-2 |
| 4-Hydroxy-benzaldehyde | Kruskal-Wallis | 1.17 (1.37)^a^ | 0.91 (0.79)^a,b^ | 0.70 (0.54)^b^ | 3.96E-2 |
| Hypoxanthine | Kruskal-Wallis | 1.00 (0.69)^a^ | 0.83 (0.56)^a^ | 0.57 (0.53)^b^ | 3.96E-2 |

Stress levels were calculated based on a psychological stress *z-*score, which was the sum of the *z-*score-transformed PSS, CDI-2, and the negative affect items from the PANAS-C. Low stress included the 25% lowest stress *z-*scores, while high stress included the 25% highest *z-*scores. Moderate stress levels included participants in the 25-75% *z-*score range. Different superscript letters (a, b, c) indicate (borderline) statistically significant pairwise differences between groups (*q≤*0.20)

Table S32b. Targeted metabolites with an overall borderline significant (*q*≤0.20) difference in the multiple comparison between stress levels in OPERA, with median QC-normalised peak area values (IQR).

| **Metabolite Name** | **Statistic** | **Low stress**  **(n=33)** | **Moderate stress**  **(n=65)** | **High stress**  **(n=33)** | ***q*** |
| --- | --- | --- | --- | --- | --- |
| N6-Acetyllysine | Kruskal-Wallis | 0.38 (0.67)^a,b^ | 0.50 (0.47)^a^ | 0.31 (0.22)^b^ | 1.03E-1 |
| 2-Picolinic acid/nicotinic acid | Kruskal-Wallis | 1.03 (0.87)^a,b^ | 1.04 (0.92)^a^ | 0.75 (0.61)^b^ | 1.03E-1 |
| Xanthine | Kruskal-Wallis | 0.79 (1.11)^a^ | 0.73 (0.85)^a^ | 0.33 (0.60)^b^ | 1.03E-1 |
| β-Hydroxyisovaleric acid | Kruskal-Wallis | 1.29 (0.70)^a^ | 1.06 (0.55)^a,b^ | 0.91 (0.45)^b^ | 2.29E-1 |
| Trimethylamine-N-oxide | Kruskal-Wallis | 0.69 (0.77) | 0.55 (0.43) | 0.49 (0.35) | 3.33E-1 |
| L-Glutamic acid | Kruskal-Wallis | 0.74 (0.64) | 0.88 (0.53) | 0.57 (0.63) | 3.34E-1 |
| Histamine | Kruskal-Wallis | 0.79 (1.39) | 0.32 (1.24) | 0.49 (1.31) | 4.37E-1 |
| 7-Methylguanine | ANOVA | 1.02 (0.60) | 1.02 (0.67) | 0.84 (0.40) | 4.37E-1 |
| 2-Ethyl-2-hydroxybutyric acid | Kruskal-Wallis | 1.23 (0.70) | 1.05 (0.58) | 0.95 (0.44) | 4.37E-1 |
| 5-Oxo-L-prolyl-L-proline | Kruskal-Wallis | 0.99 (1.94) | 0.64 (0.88) | 0.50 (1.08) | 4.37E-1 |
| N-Acetylleucine | Kruskal-Wallis | 1.10 (1.24) | 0.88 (1.07) | 0.73 (0.88) | 4.37E-1 |
| Deoxycarnitine | ANOVA | 1.05 (0.68) | 1.15 (0.69) | 0.84 (0.71) | 4.37E-1 |
| Thymine | Kruskal-Wallis | 0.78 (0.90) | 0.65 (0.58) | 0.52 (0.30) | 4.37E-1 |
| 3-Indoleacetic acid | Kruskal-Wallis | 0.94 (1.85) | 0.71 (1.40) | 0.56 (0.81) | 4.37E-1 |
| γ-Butyrolactone | Kruskal-Wallis | 0.85 (0.62) | 0.95 (0.69) | 0.73 (0.42) | 4.39E-1 |
| Acesulfame K | Kruskal-Wallis | 0.01 (0.25) | 0.00 (0.15) | 0.02 (1.00) | 4.39E-1 |
| N,N-Dimethylarginine | Kruskal-Wallis | 1.05 (0.85) | 1.06 (0.67) | 0.87 (0.80) | 4.39E-1 |
| L-Proline | Kruskal-Wallis | 0.85 (0.99) | 0.73 (0.94) | 0.54 (0.97) | 4.39E-1 |
| L-Arginine | Kruskal-Wallis | 0.78 (0.48) | 0.76 (0.35) | 0.66 (0.31) | 4.75E-1 |
| N-Acetylmethionine | Kruskal-Wallis | 0.64 (1.29) | 0.60 (0.98) | 0.41 (1.12) | 4.75E-1 |
| D-Pantothenic acid | Kruskal-Wallis | 1.06 (1.10) | 1.18 (1.49) | 0.75 (1.62) | 4.75E-1 |

Stress levels were calculated based on a psychological stress *z-*score, which was the sum of the *z-*score-transformed PSS, CDI-2, and the negative affect items from the PANAS-C. Low stress included the 25% lowest stress *z-*scores, while high stress included the 25% highest *z-*scores. Moderate stress levels included participants in the 25-75% *z-*score range. Different superscript letters (a, b, c) indicate (borderline) statistically significant pairwise differences between groups (*q≤*0.20).

Table S33. Borderline significant correlations (*q*≤0.20) of targeted salivary metabolites, related to lifestyle factors in the Fin-HIT cohort.

| **Metabolite Name** | **Lifestyle** | **Spearman ρ** | ***q*** |
| --- | --- | --- | --- |
| L-Glutamic acid | PA | 0.110 | 1.41E-1 |
| N-Acetylleucine | PA | -0.109 | 1.41E-1 |

PA: physical activity

Table S34. Borderline significant correlations (*q*≤0.20) of targeted salivary metabolites, related to lifestyle factors in the PANIC cohort.

| **Metabolite Name** | **Lifestyle** | **Spearman ρ** | ***q*** |
| --- | --- | --- | --- |
| N-Acetylleucine | PA | 0.132 | 1.15E-1 |

PA: physical activity

Table S35a (separate file). Significant correlations (*q*≤0.05) of targeted salivary metabolites, related to food consumption for the FAME cohort.

(see Excel file)

Table S35b (separate file). Borderline significant correlations (0.05<*q*≤0.20) of targeted salivary metabolites, related to food consumption for the FAME cohort.

(see Excel file)

Table S36. Borderline significant correlations (*q*≤0.20) of targeted salivary metabolites, related to food consumption for the Fin-HIT cohort.

| **Metabolite name** | **Food item** | **Spearman ρ** | ***q*** |
| --- | --- | --- | --- |
| Caffeine | Chocolate and sweets | 0.161 | 1.58E-1 |
| 2,6-Dihydroxybenzoic acid | Dark bread | 0.148 | 1.58E-1 |
| Caffeine | Soft drinks | 0.147 | 1.58E-1 |
| L-Glutamic acid | Water | 0.142 | 1.58E-1 |
| Acesulfame K | Soft drinks | 0.142 | 1.58E-1 |
| 4-Hydroxybenzaldehyde | Dark bread | -0.136 | 1.76E-1 |

Table S37a (separate file). Targeted metabolites significantly (*q*≤0.05) different between consumption levels in the FAME cohort, with median QC-normalised peak area values (IQR).

The low-consumption group comprised responses indicating consumption frequencies of ‘(almost) never’ and ‘a few times a week’, whereas the high-consumption group included responses indicating consumption frequencies ‘(almost) daily’ and ‘multiple times a day’ (see Excel file).

Table S37b (separate file). Targeted metabolites borderline significantly (0.05<*q*≤0.20) different between consumption levels in the FAME cohort, with median QC-normalised peak area values (IQR).

The low-consumption group comprised responses indicating consumption frequencies of ‘(almost) never’ and ‘a few times a week’, whereas the high-consumption group included responses indicating consumption frequencies ‘(almost) daily’ and ‘multiple times a day’ (see Excel file).

Table S38a. Targeted metabolites significantly (*q*≤0.05) different between consumption levels in the Fin-HIT cohort, with median QC-normalised peak area values (IQR).

| **Metabolite Name** | **Statistics** | **Food item** | **Low consumption** | **High consumption** | ***q*** |
| --- | --- | --- | --- | --- | --- |
| 2,6-Dihydroxy-benzoic acid | Wilcoxon | Dark bread | 0.82 (0.78) | 0.95 (0.66) | 1.96E-2 |
| 4-Hydroxy-benzaldehyde | Wilcoxon | Dark bread | 0.90 (0.50) | 0.82 (1.02) | 1.96E-2 |
| Caffeine | Wilcoxon | Dark bread | 0.28 (1.27) | 0.17 (1.04) | 2.24E-2 |
| Caffeine | Wilcoxon | Chocolate and sweets | 0.19 (0.94) | 0.55 (0.58) | 2.24E-2 |
| L-Leucine | Wilcoxon | Dark bread | 0.81 (0.66) | 0.70 (0.68) | 3.38E-2 |
| L-Glutamic acid | Wilcoxon | Fruit juice | 0.72 (0.42) | 0.80 (0.56) | 4.71E-2 |

High- and low-consumption groups were determined using a median split of the reported consumption frequencies for every food item. The low-consumption group included frequencies at or below the median, while the high-consumption group included frequencies above the median.

Table S38b. Targeted metabolites borderline significantly (0.05<*q*≤0.20) different between consumption levels in the Fin-HIT cohort, with median QC-normalised peak area values (IQR).

| **Metabolite Name** | **Statistics** | **Food item** | **Low consumption** | **High consumption** | ***q*** |
| --- | --- | --- | --- | --- | --- |
| L-Tryptophan | Wilcoxon | Fruit juice | 1.06 (0.44) | 0.96 (1.30) | 6.09E-2 |
| L-Glutamic acid | Wilcoxon | Fresh vegetables | 0.74 (0.45) | 0.81 (0.78) | 9.00E-2 |
| 3-Indoleacetic acid | Wilcoxon | Chocolate and sweets | 0.82 (1.01) | 0.57 (0.87) | 1.15E-1 |
| Thymine | Wilcoxon | Chocolate and sweets | 0.77 (0.92) | 0.63 (0.94) | 1.15E-1 |
| L-Proline | Wilcoxon | Ice cream | 0.50 (0.80) | 0.64 (0.09) | 1.30E-1 |
| Theobromine | Wilcoxon | Ice cream | 0.49 (1.64) | 0.77 (0.66) | 1.30E-1 |
| N-Acetyltryptophan | Wilcoxon | Ice cream | 1.13 (0.60) | 1.01 (1.69) | 1.30E-1 |
| Theobromine | Wilcoxon | Chocolate and sweets | 0.53 (1.58) | 0.80 (0.46) | 1.77E-1 |
| Hypoxanthine | Wilcoxon | Chocolate and sweets | 0.17 (0.97) | 0.10 (0.44) | 1.78E-1 |
| L-Glutamic acid | Wilcoxon | Water | 0.73 (0.47) | 0.80 (1.10) | 1.79E-1 |
| Theobromine | Wilcoxon | Sugary juice | 0.50 (1.45) | 0.77 (0.32) | 1.81E-1 |
| Caffeine | Wilcoxon | Sugary juice | 0.18 (0.92) | 0.43 (0.77) | 1.81E-2 |
| Theobromine | Wilcoxon | Dark bread | 0.63 (2.02) | 0.50 (0.42) | 1.88E-1 |
| Caffeine | Wilcoxon | Ice cream | 0.19 (0.99) | 0.33 (0.42) | 1.91E-1 |
| N-Acetyl-methionine | Wilcoxon | Dark bread | 0.99 (0.76) | 0.93 (0.50) | 1.98E-1 |

High- and low-consumption groups were determined using a median split of the reported consumption frequencies for every food item. The low-consumption group included frequencies at or below the median, while the high-consumption group included frequencies above the median.

Table S39a. Targeted metabolites significantly (*q*≤0.05) different between consumption levels in the OPERA cohort, with QC-normalised median peak area values (IQR).

| **Metabolite Name** | **Statistics** | **Food item** | **Low consumption** | **High consumption** | ***q*** |
| --- | --- | --- | --- | --- | --- |
| Acesulfame K | Wilcoxon | Milk | 0.13 (1.89) | 0.00 (0.08) | 4.14E-2 |

The low-consumption group comprised responses indicating consumption frequencies of ‘(almost) never’ and ‘a few times a week’, whereas the high-consumption group included responses indicating consumption frequencies ‘(almost) daily’ and ‘multiple times a day’.

Table S39b (separate file). Targeted metabolites borderline significantly (0.05<*q*≤0.20) different between consumption levels in the OPERA cohort, with QC-normalised median peak area values (IQR).

The low-consumption group comprised responses indicating consumption frequencies of ‘(almost) never’ and ‘a few times a week’, whereas the high-consumption group included responses indicating consumption frequencies ‘(almost) daily’ and ‘multiple times a day’.

Table S40. Borderline significant correlations (*q*≤0.20) of targeted salivary metabolites, related to food consumption for the PANIC cohort.

| **Metabolite name** | **Food item** | **Spearman ρ** | ***q*** |
| --- | --- | --- | --- |
| Spermidine/acetylcholine | Other beverages | 0.244 | 8.80E-2 |
| Acesulfame K | Artificially sweetened beverages | 0.228 | 1.42E-1 |

Table S41a. Targeted metabolites significantly (*q*≤0.05) different between consumption level in the PANIC cohort with QC-normalised median peak area values (IQR).

| **Metabolite Name** | **Statistics** | **Food item** | **Low consumption** | **High consumption** | ***q*** |
| --- | --- | --- | --- | --- | --- |
| Spermidine/  acetylcholine | Welch t-test | Other beverage | 1.10 (0.74) | 0.84 (0.70) | 1.54E-2 |
| Acesulfame K | Wilcoxon | Artificially sweetened beverages | 0.03 (0.23) | 0.14 (1.79) | 1.98E-2 |
| 5-Oxo-L-prolyl-L-proline | Wilcoxon | Potato | 0.45 (0.46) | 0.62 (0.63) | 3.23E-2 |
| L-Proline | Wilcoxon | Potato | 0.62 (0.54) | 0.76 (0.63) | 3.23E-2 |
| Trimethylamine-N-oxide | Wilcoxon | Jam | 0.81 (0.49) | 1.00 (0.63) | 3.55E-2 |

High- and low-consumption groups were determined using a median split of the reported consumption frequencies for every food item. The low-consumption group included frequencies at or below the median, while the high-consumption group included frequencies above the median.

Table S41b (separate file). Targeted metabolites borderline significantly (0.05<*q*≤0.20) different between consumption level in the PANIC cohort with QC-normalised median peak area values (IQR).

High- and low-consumption groups were determined using a median split of the reported consumption frequencies for every food item. The low-consumption group included frequencies at or below the median, while the high-consumption group included frequencies above the median (see Excel file).

Table S42. Consumption frequency (mean ± standard error) of diet carbonated drinks according to weight status (healthy weight *vs.* overweight *vs.* obesity) in the FAME cohort.

| **Foot item** | **Healthy weight (n=339)** | **Overweight (n=62)** | **Obese (n=39)** | ***q*** |
| --- | --- | --- | --- | --- |
| Carbonated diet drinks | 0.50 ± 0.040^c^ | 0.97 ± 0.12^b^ | 1.33 ± 0.14^a^ | 1.15E-8 |
| Savory snacks | 0.16 ± 0.44^b^ | 0.29 ± 0.55^a^ | 0.38 ± 0.71^a^ | 5.67E-3 |

Based on participants with available FFQ data (n=440), reported consumption frequencies—’(almost) never’, ‘a few times a week’, ‘(almost) daily’, and ‘multiple times per day’—were assigned numerical values of 0, 1, 2, and 3, respectively. Different superscript letters (a, b, c) indicate (borderline) statistically significant pairwise differences between groups (*q≤*0.20).

Table S43. Consumption frequency (mean ± standard error) of diet carbonated drinks according to stress status in the FAME cohort.

| **Food item** | **Low stress (n=109)** | **Moderate stress (n=224)** | **High stress (n=107)** | ***q*** |
| --- | --- | --- | --- | --- |
| Carbonated diet drinks | 0.62 ± 0.076^a,b^ | 0.54 ± 0.052^b^ | 0.88 ± 0.096^a^ | 2.61E-2 |
| Savory snacks | 0.17 ± 0.45 | 0.21 ± 0.49 | 0.19 ± 0.53 | 6.10E-1 |

Based on participants with available mental wellbeing data (n=440), reported consumption frequencies—’(almost) never’, ‘a few times a week’, ‘(almost) daily’, and ‘multiple times per day’—were assigned numerical values of 0, 1, 2, and 3, respectively. Stress levels were calculated based on a psychological stress *z-*score, which included *z-*score-transformed CDI-2 and PSS scores which were summed, and the *z-*score-transformed RSES score, which was subtracted. Low stress included the 25% lowest stress *z-*scores, while high stress included the 25% highest *z-*scores. Moderate stress levels included participants in the 25-75% *z-*score range. Different superscript letters (a, b, c) indicate (borderline) statistically significant pairwise differences between groups (*q≤*0.20).

Table S44. Consumption frequency (mean ± standard error) of diet carbonated drinks according to weight status (healthy weight *vs.* overweight *vs.* obesity) in the Fin-HIT cohort.

| **Food item** | **Healthy weight (n=234)** | **Overweight (n=189)** | **Obese (n=51)** | ***q*** |
| --- | --- | --- | --- | --- |
| Soft drinks | 2.63 ± 1.13^b^ | 2.62 ± 1.13^b^ | 3.06 ± 1.33^a^ | 1.80E-1 |
| Ice cream | 2.49 ± 1.17 | 2.30 ± 1.05 | 2.33 ± 0.74 | 3.01E-1 |

Based on participants with available FFQ data (n=474), reported consumption frequencies were recorded on a seven-point scale ranging from 0 (not consumed) to 6 (consumed several times per day), indicating the frequency of consumption for each item during the past month. Different superscript letters (a, b, c) indicate (borderline) statistically significant pairwise differences between groups (*q≤*0.20).

Table S45. Consumption frequency (mean ± standard error) of artificially sweetened beverages drinks according to weight status (healthy weight *vs.* overweight *vs.* obesity) in the PANIC cohort.

| **Food item** | **Healthy weight (n=245)** | **Overweight (n=25)** | **Obese (n=6)** | ***q*** |
| --- | --- | --- | --- | --- |
| Artificially sweetened beverages | 36.54 ± 65.00^b^ | 68.13 ± 86.85^a^ | 33.33 ± 40.82^b^ | 1.15E-1 |

Based on participants with available FFQ data (n=134), dietary intake was assessed by food records of four consecutive days that consisted of two weekdays and two weekend days. Different superscript letters (a, b, c) indicate (borderline) statistically significant pairwise differences between groups (*q≤*0.20).

Table S46. Consumption frequency (mean ± standard error) of diet drinks according to weight status (healthy weight *vs.* overweight *vs.* obesity) in the OPERA cohort.

| **Food item** | **Healthy weight (n=33)** | **Overweight (n=65)** | **Obese (n=33)** | ***q*** |
| --- | --- | --- | --- | --- |
| Diet drinks | 1.53 ± 0.85^c^ | 2.38 ± 1.35^b^ | 3.58 ± 1.78^a^ | 1.70E-6 |
| Frozen snacks | 1.42 ± 0.58^b^ | 1.72 ± 0.80^a,b^ | 2.00 ± 0.74^a^ | 1.69E-2 |

Based on participants with available FFQ data (n=134), reported consumption frequencies—’(almost) never’, ‘a few times a week’, ‘(almost) daily’, and ‘multiple times per day’—were assigned numerical values of 0, 1, 2, and 3, respectively. Different superscript letters (a, b, c) indicate (borderline) statistically significant pairwise differences between groups (*q≤*0.20).

Table S47. Consumption frequency (mean ± standard error) of diet carbonated drinks according to stress status in the Fin-HIT cohort.

| **Food item** | **Low stress (n=109)** | **Moderate stress (n=224)** | **High stress (n=107)** | ***q*** |
| --- | --- | --- | --- | --- |
| Diet drinks | 2.60 ± 1.19^b^ | 2.67 ± 1.15^b^ | 2.85 ± 1.19^a^ | 1.73E-1 |
| Ice cream | 2.41 ± 1.14 | 2.42 ± 1.09 | 2.41 ± 1.12 | 9.41E-1 |

Based on participants with available mental wellbeing data (n=474), reported consumption frequencies were recorded on a seven-point scale ranging from 0 (not consumed) to 6 (consumed several times per day), indicating the frequency of consumption for each item during the past month. Stress levels were calculated based on a psychological stress *z-*score, which included the absolute value of the *z-*score-transformed Collins Childhood Body Rating Scale score, with the *z-*score of RSES subtracted. Low stress included the 25% lowest stress *z-*scores, while high stress included the 25% highest *z-*scores. Moderate stress levels included participants in the 25-75% *z-*score range. Different superscript letters (a, b, c) indicate (borderline) statistically significant pairwise differences between groups (*q≤*0.20).

Table S48. Consumption frequency (mean ± standard error) of diet carbonated drinks according to stress status in the OPERA cohort.

| **Food item** | **Low stress (n=193)** | **Moderate stress (n=29)** | **High stress (n=12)** | ***q*** |
| --- | --- | --- | --- | --- |
| Diet drinks | 1.88 ± 1.02 | 1.77 ± 1.17 | 2.09 ± 1.51 | 7.60E-1 |
| Frozen snacks | 1.48 ± 0.62 | 1.57 ± 0.64 | 1.63 ± 0.78 | 7.60E-1 |

Based on participants with available mental wellbeing data (n=134), reported consumption frequencies—’(almost) never’, ‘a few times a week’, ‘(almost) daily’, and ‘multiple times per day’—were assigned numerical values of 0, 1, 2, and 3, respectively. Stress levels were calculated based on a psychological stress *z-*score, which included *z-*score-transformed CDI-2 and PSS scores, which were summed, and the *z-*score-transformed RSES score, which was subtracted. Low stress included the 25% lowest stress *z-*scores, while high stress included the 25% highest *z-*scores. Moderate stress levels included participants in the 25-75% *z-*score range. Different superscript letters (a, b, c) indicate (borderline) statistically significant pairwise differences between groups (*q≤*0.20).

Table S49. Consumption frequency (mean ± standard error) of artificially sweetened beverages according to stress status in the PANIC cohort.

| **Food item** | **Low stress (n=88)** | **Moderate stress (n=130)** | **High stress (n=49)** | ***q*** |
| --- | --- | --- | --- | --- |
| Artificially sweetened beverages | 43.77 ± 73.69 | 31.17 ± 53.19 | 54.61 ± 87.12 | 2.91E-1 |

Based on participants with available mental wellbeing data (n=267). Dietary intake was assessed by food records of four consecutive days that consisted of two weekdays and two weekend days. Stress levels were calculated based on sleep quality. Low stress included the 25% lowest stress *z-*scores, while high stress included the 25% highest *z-*scores. Moderate stress levels included participants in the 25-75% *z-*score range. Different superscript letters (a, b, c) indicate (borderline) statistically significant pairwise differences between groups (*q≤*0.20).

Table S50a (separate file). Significant correlations (*q≤*0.05) between the relative abundance of salivary bacteria and targeted salivary metabolites in the Fin-HIT cohort.

(see Excel file)

Table S50b (separate file). Borderline significant correlations (0.05<*q≤*0.20) between the relative abundance of salivary bacteria and targeted salivary metabolites in the Fin-HIT cohort.

(see Excel file)

Table S51a (separate file). Significant correlations (*q≤*0.05) between the relative abundance of salivary bacteria and targeted salivary metabolites in the PANIC cohort.

(see Excel file)

Table S51b (separate file). Borderline significant correlations (0.05<*q≤*0.20) between the relative abundance of salivary bacteria and targeted salivary metabolites in the PANIC cohort.

(see Excel file)

1. These authors contributed equally to this work. [↑](#footnote-ref-2)
